# Acceptability, Tolerability, and Estimates of Putative Treatment Effects of Probiotics as Adjunctive Treatment in Patients With Depression: A Randomized Clinical Trial

**DOI:** 10.1001/jamapsychiatry.2023.1817

**Published:** 2023-06-14

**Authors:** Viktoriya L. Nikolova, Anthony J. Cleare, Allan H. Young, James M. Stone

**Affiliations:** 1 Centre for Affective Disorders, Institute of Psychiatry, Psychology & Neuroscience, King’s College London, London, United Kingdom; 2 ADM Health & Wellness, ADM Protexin, Somerset, United Kingdom; 3 National Institute for Health Research Biomedical Research Centre at South London and Maudsley NHS Foundation Trust and King’s College London, London, United Kingdom; 4 South London and Maudsley NHS Foundation Trust, Bethlem Royal Hospital, Beckenham, United Kingdom; 5 Brighton and Sussex Medical School, Brighton, United Kingdom

## Abstract

**Importance:**

The microbiota-gut-brain axis is a promising target for novel treatments for mood disorders, such as probiotics. However, few clinical trials have been conducted, and further safety and efficacy data are needed to support this treatment approach.

**Objective:**

To provide acceptability and tolerability data and estimates of intervention effect size for probiotics as adjunctive treatment for patients with major depressive disorder (MDD).

**Design, Setting, and Participants:**

In this single-center, double-blind, placebo-controlled pilot randomized clinical trial, adults aged 18 to 55 years with MDD taking antidepressant medication but having an incomplete response were studied. A random sample was recruited from primary and secondary care services and general advertising in London, United Kingdom. Data were collected between September 2019 and May 2022 and analyzed between July and September 2022.

**Intervention:**

Multistrain probiotic (8 billion colony-forming units per day) or placebo daily for 8 weeks added to ongoing antidepressant medication.

**Main Outcomes and Measures:**

The pilot outcomes of the trial were retention, acceptability, tolerability, and estimates of putative treatment effect on clinical symptoms (depression: Hamilton Depression Rating Scale [HAMD-17] and Inventory of Depressive Symptomatology [IDS] scores; anxiety: Hamilton Anxiety Rating Scale [HAMA] and General Anxiety Disorder [GAD-7] scores) to be used as indicators for a definitive trial.

**Results:**

Of 50 included participants, 49 received the intervention and were included in intent-to-treat analyses; of these, 39 (80%) were female, and the mean (SD) age was 31.7 (9.8) years. A total of 24 were randomized to probiotic and 25 to placebo. Attrition was 8% (1 in the probiotic group and 3 in the placebo group), adherence was 97.2%, and there were no serious adverse reactions. For the probiotic group, mean (SD) HAMD-17 scores at weeks 4 and 8 were 11.00 (5.13) and 8.83 (4.28), respectively; IDS, 30.17 (11.98) and 25.04 (11.68); HAMA, 11.71 (5.86) and 8.17 (4.68); and GAD-7, 7.78 (4.12) and 7.63 (4.77). For the placebo group, mean (SD) HAMD-17 scores at weeks 4 and 8 were 14.04 (3.70) and 11.09 (3.22), respectively; IDS, 33.82 (9.26) and 29.64 (9.31); HAMA, 14.70 (5.47) and 10.95 (4.48); and GAD-7, 10.91 (5.32) and 9.48 (5.18). Standardized effect sizes (SES) from linear mixed models demonstrated that the probiotic group attained greater improvements in depressive symptoms according to HAMD-17 scores (week 4: SES, 0.70; 95% CI, 0.01-0.98) and IDS Self Report scores (week 8: SES, 0.64; 95% CI, 0.03-0.87) as well as greater improvements in anxiety symptoms according to HAMA scores (week 4: SES, 0.67; 95% CI, 0-0.95; week 8: SES, 0.79; 95% CI, 0.06-1.05), but not GAD-7 scores (week 4: SES, 0.57; 95% CI, −0.01 to 0.82; week 8: SES, 0.32; 95% CI, −0.19 to 0.65), compared with the placebo group.

**Conclusions and Relevance:**

The acceptability, tolerability, and estimated effect sizes on key clinical outcomes are promising and encourage further investigation of probiotics as add-on treatment for people with MDD in a definitive efficacy trial.

**Trial Registration:**

ClinicalTrials.gov Identifier: NCT03893162

## Key Points

**Question:**

Are probiotics an acceptable, tolerable, and potentially efficacious adjunctive treatment for depression?

**Findings:**

In this pilot randomized clinical trial including 49 participants, daily probiotic intake for 8 weeks resulted in greater improvements in depressive and anxiety symptoms compared with placebo.

**Meaning:**

The acceptability, tolerability, and estimated effect sizes on key clinical outcomes are promising and encourage further investigation of probiotics as adjunctive treatment for people with major depressive disorder in a definitive efficacy trial.

## Teaser

This pilot randomized clinical trial evaluates acceptability and tolerability data and estimates of intervention effect size for probiotics as adjunctive treatment for patients with major depressive disorder.

## Introduction

Approximately 60% of people with major depressive disorder (MDD) experience some degree of nonresponse to first-line treatments, and approximately one-third continue to experience symptoms despite further treatment.^1^ Increasing understanding of the involvement of the microbiota-gut-brain axis in the pathophysiology of MDD has made it a promising target for novel treatments, such as probiotics.

In a 2021 meta-analysis of 7 randomized clinical trials (RCTs) including 404 patients,^2^ we found that probiotics appear to be effective in reducing depressive symptoms when administered adjunctively to antidepressants. However, several RCTs to date have not provided sufficient tolerability data, while others report poor adherence or retention.^2,3^ Therefore, further safety and efficacy data are needed for probiotics to be considered a viable treatment option in clinical practice.

## Methods

This study was part of a larger 8-week randomized double-blind placebo-controlled RCT. The aims of the main trial were mechanistic; however, the evaluation of the feasibility/pilot outcomes presented here was part of the protocol approved by the London-Surrey Research Ethics Committee. Written informed consent was obtained from all participants. The trial protocol can be found in Supplement 1. This study followed the Consolidated Standards of Reporting Trials (CONSORT) reporting guideline.

### Participants

A total of 50 outpatients with a primary diagnosis of MDD and with a Hamilton Depression Rating Scale (HAMD-17)^4^ score greater than 13 were recruited. All participants were taking an approved antidepressant at a stable dose for 6 or more weeks and were required to not make changes throughout the study. Exclusion criteria were bipolar disorder, psychosis, eating disorders, personality disorders, substance dependence, or suicidal ideation; serious medical illness, gastrointestinal disease or surgery; use of antibiotics or probiotics in the past 12 weeks; current or regular gastrointestinal medication use; smoking; pregnancy or breastfeeding; and a vegan diet. Race and ethnicity data were collected through self-report by choosing one of the following categories: Asian (non-Chinese; British or any other), Black (British or any other), Chinese, multiracial (any), White (British or any other), or other race (any).

### Intervention and Blinding

Participants were randomized 1:1 to 4 capsules daily of probiotic (2 × 10^9^ colony-forming units per capsule) or matching placebo. The probiotic contained 14 strains of *Bacillus subtilis*, *Bifidobacterium bifidum*, *Bifidobacterium breve*, *Bifidobacterium infantis*, *Bifidobacterium longum*, *Lactobacillus acidophilus*, *Lactobacillus delbrueckii subsp bulgaricus*, *Lactobacillus casei*, *Lactobacillus plantarum*, *Lactobacillus rhamnosus*, *Lactobacillus helveticus*, *Lactobacillus salivarius*, *Lactococcus lactis*, and *Streptococcus thermophilus* (Bio-Kult Advanced; ADM Protexin) and was selected due to earlier evidence of antidepressant effects of these species.^2,5^ Success of masking was evaluated by asking participants to guess their allocation at study end. For detailed intervention, randomization, and blinding procedures, see eMethods in Supplement 2.

### Procedure and Outcomes

Participants attended 3 visits (baseline, week 4, and week 8). The primary outcome for a future efficacy RCT was change in depressive scores at week 8 (HAMD-17 and Inventory of Depressive Symptomatology [IDS] Self Report).^6^ Other outcomes included changes in anxiety (Hamilton Anxiety Rating Scale [HAMA]^7^ and General Anxiety Disorder [GAD-7] scores)^8^ and clinical status (Clinical Global Impression [CGI])^9^ scores as well as adherence. Adverse events and gastrointestinal symptoms were also monitored (eMethods in Supplement 2).

### Statistical Analysis

We recruited 50 participants, consistent with recommendations for pilot studies aiming to perform power calculations.^10,11^ Estimates of efficacy were calculated on the intent-to-treat (ITT) and per-protocol (PP) principles and measured the effect size of the between-group mean difference. Linear mixed models were performed with the outcomes as the dependent variables, treatment group, time, and time × group interaction as the fixed terms, and random intercept for participant. Standardized effect sizes (SES) with small sample size correction were calculated. For CGI Severity, as an ordinal outcome, generalized linear testing (ordinal logistic) was performed. CGI Improvement scores were analyzed with χ^2^ likelihood ratio tests. Analyses were performed in SPSS version 28 (IBM), with 2-tailed significance level set at *P* < .05. The confounding effects of body mass index, age, weight, gastrointestinal complaints, alcohol intake, and dietary parameters were evaluated. Further details can be found in eMethods in Supplement 2.

## Results

Of 50 included participants, 49 received the intervention and were included in intent-to-treat analyses; of these, 39 (80%) were female, and the mean (SD) age was 31.7 (9.8) years. A total of 24 were randomized to probiotic and 25 to placebo. Three further participants in the placebo group dropped out, resulting in 46 completers and an attrition rate of 8%. Two participants commenced antibiotics and were included in the ITT analysis but not PP analysis (eFigure 1 in Supplement 2).

Participant characteristics are in Table 1. Baseline depression severity was moderate, and 45 of 49 participants (92%) were taking a selective serotonin-reuptake inhibitor (SSRI). Anxiety comorbidities were common, with 21 (43%) meeting criteria for generalized anxiety disorder. The only difference between groups was race, with all participants identifying as Asian (non-Chinese) allocated to probiotics (7 of 24 [29%] vs 0 of 25).

**Table 1.** Baseline Demographic and Clinical Characteristics by Treatment Group

Characteristic	No. (%)	
	Probiotic group (n = 24)	Placebo group (n = 25)
Age, median (IQR), y	32.5 (24.3-39.0)	27.0 (23.0-41.0)
Sex		
Female	18 (75)	21 (84)
Male	6 (25)	4 (16)
Weight, mean (SD), kg	72.1 (18.5)	79.7 (22.1)
BMI, median (IQR)^a^	23.3 (21.3-29.2)	25.9 (23.9-31.9)
Race and ethnicity^b^		
Asian (non-Chinese)	7 (29)	0
Multiracial	3 (12)	3 (12)
White	13 (54)	20 (80)
Other race	1 (4)	2 (8)
Depression severity		
HAMD-17 total score, mean (SD)	16.5 (2.9)	17.3 (3.2)
IDS-SR total score, mean (SD)	37.5 (10.1)	37.0 (6.3)
CGI-S, median (IQR)	4.0 (3.0-4.0)	4.0 (4.0-4.0)
Duration of episode, median (IQR), wk	51.0 (22.5-103.3)	55.0 (22.5-173.0)
Number of episodes, median (IQR)	3.0 (1.0-3.0)	3.0 (1.0-5.0)
Antidepressant treatment		
Duration, median (IQR), wk	59.0 (11.0-201.0)	34.0 (11.0-97.0)
Class		
SSRI	21 (88)	24 (96)
SNRI	3 (13)	0
Other	0	1 (4)
Anxiety comorbidity		
None	12 (50)	9 (36)
Generalized anxiety disorder	10 (42)	11 (44)
Any other anxiety disorder	8 (33)	10 (40)
OCD	0	1 (4)
HAMA total score, mean (SD)	15.8 (4.7)	16.1 (5.1)
GAD-7 total score, mean (SD)	10.6 (4.0)	11.0 (4.6)
Diet and gastrointestinal health		
Vegetarian	3 (13)	4 (16)
GSRS total score, median (IQR)	12.5 (7.3-20.5)	11.0 (8.0-22.0)
FFQ dietary quality, median (IQR), g per wk		
Fruit	20.0 (11.2-56.8)	56.8 (11.2-56.8)
Vegetables	56.8 (28.8-81.2)	68.0 (36.4-127.6)
Oily fish	0 (0-10.6)	0 (0-2.3)
Fat	30.9 (19.7-46.6)	34.8 (20.4-47.8)
NMES	21.1 (10.6-35.0)	21.6 (10.9-38.8)
Alcohol intake		
Rarely/never drink	11 (46)	9 (36)
<14 Units per wk	8 (33)	13 (52)
14-21 Units per wk	4 (17)	3 (12)
>21 Units per wk	1 (4)	0

Abbreviations: CGI-S, Clinical Global Impression Severity subscale; FFQ, Food Frequency Questionnaire, Short-Form; GAD-7, General Anxiety Disorder; GSRS, Gastrointestinal Symptom Rating Scale; HAMA, Hamilton Anxiety Rating Scale; HAMD-17, Hamilton Depression Rating Scale, 17-item; IDS-SR, Inventory of Depressive Symptomatology Self Report; NMES, non-milk extracted sugars; OCD, obsessive-compulsive disorder; SNRI, serotonin–norepinephrine reuptake inhibitors; SSRI, selective serotonin reuptake inhibitors.

^a^ Calculated as weight in kilograms divided by height in meters squared.

^b^ Race and ethnicity data were collected through self-report by choosing one of the following categories: Asian (non-Chinese; British or any other), Black (British or any other), Chinese, multiracial (any), White (British or any other), or other race (any). Due to low numbers, the Black, Chinese and other race categories were combined.

### Adherence, Tolerability, and Blinding

The masking was successful, with a nonsignificant correct guess rate between groups (8 [33%] in the probiotic group and 5 [23%] in the placebo group) and 22 participants (48%) selecting that they did not know (eTable 1 in Supplement 2). This rate is likely low due to the appropriate concealment and low adverse-effect profile of the intervention. Adherence was high, with 97.2% of doses taken as required (capsule count). The intervention was well tolerated, with no serious adverse reactions and no dropouts owing to adverse effects. A total of 16 participants reported adverse reactions (eTable 2 in Supplement 2). Of these, nausea and indigestion were experienced only in the probiotic group but were transient and did not require medication. Gastrointestinal symptom scores decreased in both groups over time and were not significant between groups (eFigure 2 in Supplement 2).

### Estimates of Putative Treatment Effects

Depressive symptoms improved in both arms, with greater reductions in the probiotic group from week 4 (Figure). A strong association between treatment group and HAMD-17 scores was observed at week 4 (SES, 0.70; 95% CI, 0.01-0.98), IDS scores at week 8 (SES, 0.64; 95% CI, 0.03-0.87), and HAMA scores at both time points (week 4: SES, 0.67; 95% CI, 0-0.95; week 8: SES, 0.79; 95% CI, 0.06-1.05) but not GAD-7 scores (week 4: SES, 0.57; 95% CI, −0.01 to 0.82; week 8: SES, 0.32; 95% CI, −0.19 to 0.65) (Table 2). None of the covariates affected findings (data not shown), nor did sensitivity analyses evaluating the impact of non-SSRI medications or the clustering of Asian (non-Chinese) individuals in the probiotic arm (eTables 3 and 4 in Supplement 2). There were no notable differences between ITT and PP data sets (eTable 5 and eFigure 3 in Supplement 2). The probiotic group also showed stronger response on the CGI Improvement subscale (Figure) but not the CGI Severity subscale (week 4: odds ratio, 0.28; 95% CI, −0.85 to 1.42; week 8: odds ratio, 0.20; 95% CI, −0.99 to 1.39).

**Figure.**
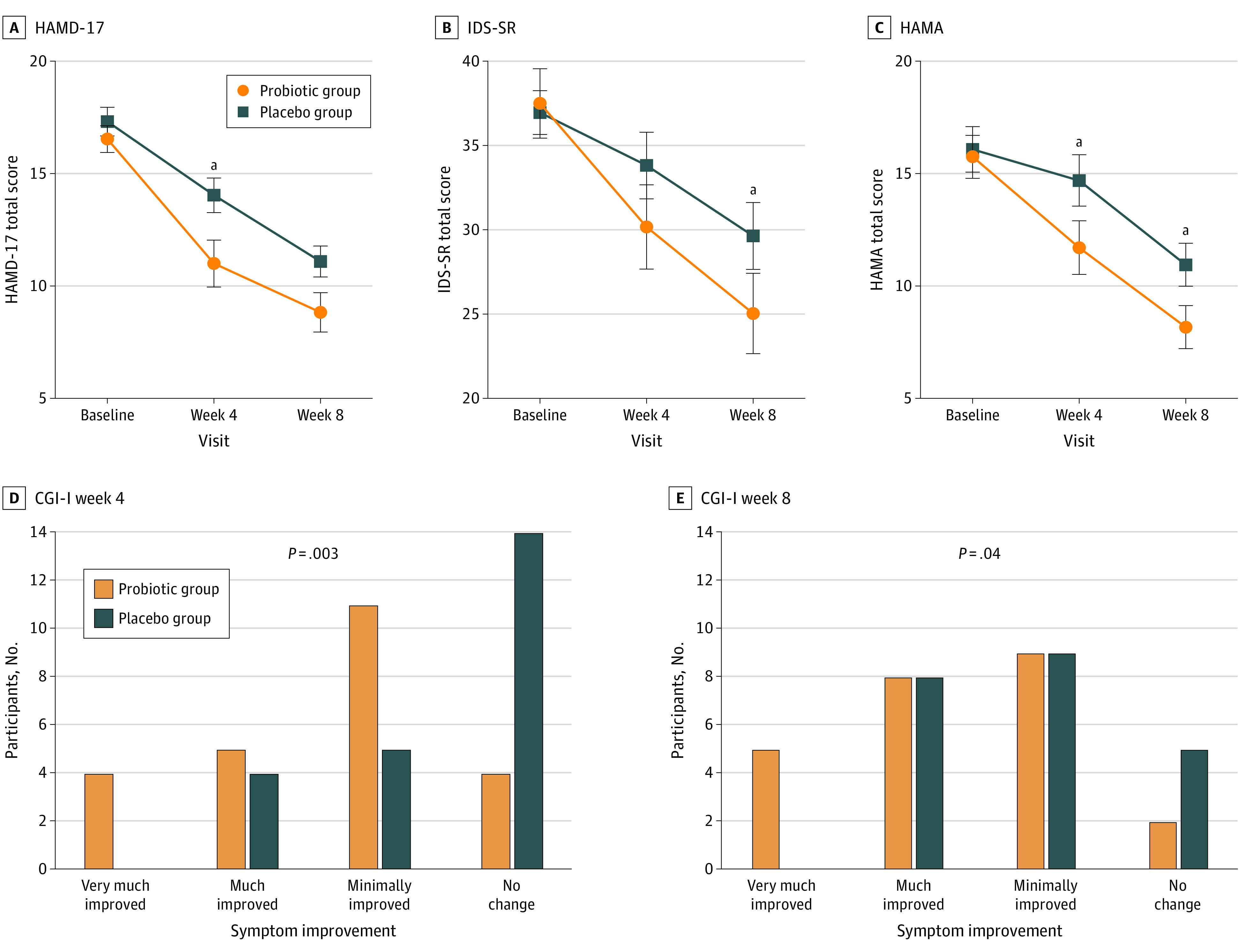
Trajectories of Depression and Anxiety Symptoms in the Probiotic and Placebo Groups Data from the intent-to-treat data set (n = 49) were analyzed. A-C, Data are presented as mean scores. Error bars indicate SE. CGI-I indicates Clinical Global Impression Improvement subscale; HAMA, Hamilton Anxiety Rating Scale; HAMD-17, Hamilton Depression Rating Scale; IDS-SR, Inventory of Depressive Symptomatology Self Report subscale. ^a^*P* < .05. As this was a pilot study, indicators of significance are included only for ease of interpretation.

**Table 2.** Estimates of Treatment Effect on Depression and Anxiety Symptoms in the Intent-to-Treat Samples

Measure	Mean (SD)		Interaction estimate (95% CI)	*t* Value	*P* value	Cohen *d* (95% CI)	Corrected Cohen *d*
	Probiotic group	Placebo group					
HAMD-17							
Week 4	11.00 (5.13)	14.04 (3.70)	2.16 (0.06 to 4.26)	2.06	.04	0.70 (0.01 to 0.98)	0.68
Week 8	8.83 (4.28)	11.09 (3.22)	1.48 (−0.77 to 3.73)	1.32	.19	0.48 (−0.25 to 1.22)	0.47
IDS-SR							
Week 4	30.17 (11.98)	33.82 (9.26)	4.14 (−0.60 to 8.87)	1.76	.09	0.49 (−0.05 to 0.74)	0.47
Week 8	25.04 (11.68)	29.64 (9.31)	5.35 (0.38 to 10.32)	2.17	.04	0.64 (0.03 to 0.87)	0.61
HAMA^a^							
Week 4	11.71 (5.86)	14.70 (5.47)	0.41 (0 to 0.82)	2.02	.05	0.67 (0 to 0.95)	0.65
Week 8	8.17 (4.68)	10.95 (4.48)	0.48 (0.05 to 0.90)	2.26	.03	0.79 (0.06 to 1.05)	0.76
GAD-7							
Week 4	7.78 (4.12)	10.91 (5.32)	2.47 (−0.07 to 5.00)	1.96	.06	0.57 (−0.01 to 0.82)	0.55
Week 8	7.63 (4.77)	9.48 (5.18)	1.40 (−1.16 to 3.96)	1.10	.28	0.32 (−0.19 to 0.65)	0.31

^a^ Interaction estimates based on square root–transformed values due to nonnormally distributed data and residuals; interaction estimates show the time group mean difference from the linear mixed models, with positive values indicating a larger improvement in the probiotic group.

### Exploratory Analyses

It has been suggested that probiotics may be beneficial as adjunctive treatment as they may help alleviate presentations that antidepressants are less effective against (eg, anxious, somatic).^2,5^ To explore this, we analyzed the 9-item Anxiety/Arousal IDS subscale^12^ using the PP data set at week 8 (eTable 6 in Supplement 2). We found significant effects, with a similar effect size to that of the main scale (SES, 0.75; 95% CI, 0.08-1.44), suggesting that the reduction in total scores may be driven by anxious and somatic symptoms.

## Discussion

To our knowledge, this is the first trial in a Western population to demonstrate the safety, acceptability, and therapeutic potential of a readily available and scalable probiotic intervention in patients with MDD. Compared with the placebo group, the probiotic group exhibited greater improvement in depressive symptoms with moderate effect sizes, which are comparable with those reported in earlier meta-analyses.^2,13^ Participants in the probiotic arm experienced, on average, a reduction of 1 severity grade on both depression rating scales.

Anxiety symptoms have been little examined in probiotic trials in depression, despite their high prevalence in MDD (approximately 40% to 50%).^14^ In addition to the greater effects on clinician-rated anxiety, our exploratory analyses suggested that anxious-somatic symptoms may have been particularly improved by the probiotic. If confirmed in larger trials, these findings could provide an indication of which patients may benefit most from probiotic treatment.

The probiotic was well tolerated, with a low attrition rate, high adherence rate, and no serious adverse reactions. This safety and acceptability profile is better than those reported in earlier studies using different supplements.^2,3^ Given the range of supplements available, clinical decisions should be guided not only by indicators of efficacy but also by safety and acceptability.

### Limitations

A limitation of this study is that we cannot ascertain whether the observed effects are specific to the interaction with SSRIs or generalizable to other treatments. Further, adherence was evaluated through capsule count, which, while the most commonly used method in clinical trials, can lead to overreporting. Nevertheless, this is the method used in most trials against which we compared adherence.

## Conclusions

In summary, the preliminary findings from this pilot study suggest that 8-week adjunctive treatment with a multistrain probiotic is acceptable and tolerable for adults with MDD. The estimated effect sizes on key clinical outcomes are promising and encourage further investigation in a definitive efficacy trial.

## Notes

Supplement 1. Trial Protocol

Supplement 2.
eMethods.
**eFigure 1.** CONSORT Diagram of Participant Flow for the PROMEX Study **eFigure 2.** GI Symptoms (GSRS9) in the Probiotic (Green) and Placebo (Blue) Groups Through the Course of Treatment **eFigure 3.** Trajectories of Depressive and Anxiety Symptoms in the Probiotic (Green) and Placebo (Blue) Groups Through the Course of the Study **eTable 1.** Success of Blinding Check **eTable 2.** Adverse Reactions Experienced by Participants in Either Group **eTable 3.** Sensitivity Analysis by Race **eTable 4.** Sensitivity Analysis by Non-SSRI Use **eTable 5.** Estimates of Treatment Effect on Depressive and Anxiety Symptoms in PP Dataset **eTable 6.** IDS Items Included in the Rush (1966) Anxiety/Arousal Subscale
eReferences.

Supplement 3. Data Sharing Statement
