# Supplementary material for: Acceptability, Tolerability, and Estimates of Putative Treatment Effects of Probiotics as Adjunctive Treatment in Patients With Depression: A Randomized Clinical Trial
Source: JAMA Psychiatry. 2023 Jun 14;80(8):842–7. doi: 10.1001/jamapsychiatry.2023.1817 (PMC10267847; doi:10.1001/jamapsychiatry.2023.1817)
Supplement: Supplement 1. — Trial Protocol [file jamapsychiatry-e231817-s001.pdf]

NHS South London and Maudsley NHS Foundation Trust

Institute of Psychiatry, Psychology & Neuroscience — KING'S College LONDON

PROTOCOL FULL TITLE

GUT FEELING: UNDERSTANDING THE MECHANISMS UNDERLYING THE ANTIDEPRESSANT PROPERTIES OF PROBIOTICS

Protocol Acronym: **PROMEX**

Co-Sponsors

**Name:**	King's College London (KCL) /South London and Maudsley NHS Foundation Trust (SLaM)		
**Contact KCL:**	Professor Reza Razavi	**Contact SLAM:**	Dr Carol Cooley
**Address:**	Room 5.31, James Clerk Maxwell Building, 57 Waterloo Rd, London, SE1 8WA	**Address:**	Room W1.08 Institute of Psychiatry, Psychology & Neuroscience, De Crespigny Park, London, SE5 8AF
**Telephone:**	0207 8483224	**Telephone:**	020 7848 0675
**Email:**	reza.razavi@kcl.ac.uk	**Email:**	slam-ioppn.research@kcl.ac.uk

Chief Investigator

**Name:**	Dr James Stone
**Address:**	Trafford Centre, University of Sussex, Falmer, Brighton BN1 9RH
**Telephone:**	01273 873833
**Fax:**	020 7848 0298
**Email:**	james.m.stone@kcl.ac.uk/ j.stone@bsms.ac.uk

Name and address of Co-Investigator(s), Laboratories etc

Name:	Viktoriya Nikolova
Position/ Role:	PhD student
Address:	Centre for Affective Disorders, Institute of Psychiatry, Psychology & Neuroscience, King's College London, London, UK.
Telephone:	07707833422
Email:	viktoriya.nikolova@kcl.ac.uk

Name:	Professor Anthony Cleare
Position/ Role:	Co-Investigator
Address:	103 Denmark Hill, Institute of Psychiatry, Psychology & Neuroscience, King's College London, London, UK
Telephone:	02078485130
Fax:	02078480783
Email:	anthony.cleare@kcl.ac.uk

Name:	Professor Allan Young
Position/ Role:	Principal Investigator, SLaM/IoPPN site
Address:	Centre for Affective Disorders, Institute of Psychiatry, Psychology & Neuroscience, King's College London, London, UK.
Telephone:	02078485895
Email:	allan.young@kcl.ac.uk

Name:	Dr Lindsay Hall
Position/ Role:	Co-Investigator, Microbiology expert
Address:	Gut Microbes & Health, Quadram Institute Bioscience, Norwich Research Park, Norwich, UK
Telephone:	01603 255167
Email:	Lindsay.Hall@quadram.ac.uk

Name:	Dr Richard Day
Position/ Role:	Collaborator (Protexin)
Address:	ADM Protexin Ltd, Lopen Head, Somerset, TA13 5JH, UK
Telephone:	01460 243 230
Email:	richard.day@adm.com

Name:	Martin Heasman
Position/ Role:	Pharmacist
Address:	Pharmacy Department, Maudsley Hospital, Denmark Hill, London, UK, SE5 8AZ
Telephone:	020 3228 5029
Fax:	020 3228 2337
Email:	Martin.Heasman@slam.nhs.uk

Name:	Dr Gursharan Kalsi,
Position/ Role:	Research Coordinator & Manager, BioResource for Mental and Neurological Health
Address:	MRC SGDP Centre, Institute of Psychiatry, Psychology and Neuroscience & SLaM NHS Trust, King's College London ,Denmark Hill, London, SE5 8AF
Telephone:	020 7848 0573
Email:	gursharan.kalsi@kcl.ac.uk

Study Synopsis

Full Title	Gut feeling: understanding the mechanisms underlying the antidepressant properties of probiotics
Acronym	PROMEX
Protocol Version and Date	Version 7.4 08Nov2021
Study Duration	37 months
Study Type	Placebo-Controlled Clinical Investigation
Study Design	Double-blind placebo-controlled parallel groups design to investigate the effects of 8 weeks of treatment with probiotics on the gut microbiota, levels of glutamate and GABA in the brain and pro-inflammatory cytokines in patients with major depressive disorder (MDD). To investigate the differences in the gut microbiota between patients with MDD and healthy individuals, the baseline samples collected in this study will be compared with samples from demographically-matched healthy volunteers (HV).
Sponsor/Co-sponsors	King's College London (KCL) /South London and Maudsley NHS Foundation Trust (SLaM)
Chief Investigator	Dr James Stone
REC number	19/LO/0761
Primary objectives	1. to characterise the gut microbiota in MDD patients;<br>2. to investigate gut microbiota differences between MDD and HV<br>3.to analyse the changes to the gut microbiota in MDD patients induced by probiotic treatment and their correlation to change in depressive symptoms at week 8
Secondary objectives	1. to investigate whether there are changes in blood levels of inflammatory cytokines after 8 weeks of probiotic treatment and whether these mediate the effects of probiotics on mood;<br>2. to investigate whether there are changes in glutamate and GABA levels in the brain after 8 weeks of probiotic treatment;<br>3.to investigate whether probiotics affect response to emotional faces (in terms of accuracy of recognition and brain activity);
Number of Subjects	50 MDD, 25 HV
Main Inclusion Criteria	For MDD: 18-55yrs, currently in a depressive episode and on a stable antidepressant; For HV: 18-55yrs, with no psychiatric history.
Statistical Methodology and Analysis	Stool samples will be subjected to microbiome analysis. Species diversity at the level of organisational taxonomic units (OTUs) will be investigated and the significance of any differences between MDD patients and healthy controls will be tested using series of Student's t-tests. Differences in the change of the microbiome of MDD patients following probiotic vs. placebo treatment will also be analysed with Student t-tests. Associations between change in bacterial species levels and change in rating scale scores as well as secondary outcome measures and covariates will be tested using General Linear Model (MANCOVA). Effect sizes will be calculated for all analyses.

Key Abbreviations

AE – Adverse Event

BDNF – brain derived neurotrophic factor

CRP – C-reactive protein

CFU – colony forming unit

FFQ – food frequency questionnaire

fMRI – functional Magnetic Resonance Imaging

HPA - hypothalamus-pituitary-adrenal [axis]

IAPT - Improving Access to Psychological Therapies

IL-1β/IL-6 – interleukin 1β/6

LPS - lipopolysaccharide

MDD – major depressive disorder

MRS - magnetic resonance spectroscopy

OUT - organisational taxonomic unit

PCR - Polymerase chain reaction

PO/SO/TO – primary/secondary/tertiary objective

QIB – Quadram Institute Bioscience

rRNA - ribosomal RNA

SAE – Serious Adverse Event

SOP – Standard Operating Procedure

TNFα – Tumour Necrosis Factor α

Contents

Sponsor 1
Chief Investigator 1
Name and address of Co-investigator(s), laboratories etc 2
Study synopsis 3
Key abbreviations 4
1. Background & rationale 7
2. Study objectives and design 9
2.1. Study objectives 9
2.2. Study design 10
2.3. Flowchart of the double-blind study 11
3. Sample size and statistics 12
3.1. Power calculation and sample size 12
3.2. Statistical analysis 12
4.1. Selection and withdrawal of participants 12
Recruitment 12
Inclusion criteria for healthy volunteers 13
Inclusion criteria for MDD participants 13
Exclusion criteria for MDD participants 13
Lifestyle guidelines (MDD participants only) 14
Withdrawal 14
4.2. Randomisation and blinding 14
4.3. Intervention 14
4.4. Concomitant medication/therapy 15
4.5. Study procedures schedule by visit 15
Informed consent procedure 18
Screening visit 18
Baseline visit 18
Phone assessments (week 2, week 6) 19
Interim visit (week 4) 19
End of treatment visit (week 8) 19
Neuroimaging sub-study 19

Emotional faces fmri task 20
Neuroimaging data analysis 20
Emotional faces fmri task data analysis 20
4.6. End of study definition 20
5. Sample handling, analysis and laboratories 21
5.1. Stool samples 21
analysis of samples 21
5.2. Blood samples handling & analysis 22
6. Assessment of safety 22
7. Impact of COVID-19 22
8. Ethics and regulatory approvals 23
9. Data handling 23
10. Dissemination 23
11. Finance 23
12. References 23

1.Background & Rationale

Depression or major depressive disorder (MDD) is a common complex and heterogeneous illness that significantly diminishes quality of life (Otte et al., 2016) and is characterised by low mood, anhedonia, sleep disturbances and self-harm or suicidal ideations. It is estimated that 216 million people suffered from MDD worldwide in 2015 (GBD, 2016) and it is projected that rates will increase due to an apparent increase in incidence rates in younger cohorts (Greenberg et al., 2015). MDD is one of the largest single leading causes of health related disability and the most costly of all mental health disorders (Prince et al., 2007). According to a recent commissioned review, the total cost of services for depression in England in 2007 was estimated at £1.7 billion and overall societal costs at £7.5 billion, accounting for the costs associated with lost employment. By 2026 these figures are projected to reach £3 billion and £12.2 billion, respectively (McCrone et al., 2008). It has been estimated that up to 60% of patients with MDD may experience some degree of treatment non-response (Fava, 2003) and about a third are considered to be treatment-resistant.

In recent years, an evidence base from pre-clinical models has accumulated demonstrating that the gut microbiota can influence gut-brain communication, brain function and behaviour. Studies with germ-free mice (with no gut bacteria) have shown that these animals exhibit increased stress response and anxiety-like behaviours and deficits in social behaviour and memory – all of which are prominent features of depressive disorders (Rios et al., 2017). These findings have also been replicated in mice given a mixture of antibiotics (Bercik et al., 2011) or with induced bacterial infection (Gareau et al., 2011). These effects have been shown to be reversed by restoring commensal microbiota or by treatment with probiotics (Gareau et al., 2011; Bercik et al., 2011; Rios et al., 2017). The reverse strategy has also been used to demonstrate the link between mood and the microbiota: by inducing depressive-like behaviours in mice and observing their effects on the microbiota through analysis of stool samples. For example, a study by Barseghyan et al. (2013) showed significantly increased numbers of *C. albicans* and *S. aureus* while number of *Lactobacilli* and *Bifidobacteria* were significantly decreased.

Few studies have been conducted to investigate human stool samples from patients with depressive symptoms. One such study by Naseribafrouei et al. (2014) investigated the relationship between faecal microbiota and major depressive disorder (MDD) using 16S ribosomal RNA (rRNA) sequencing and discovered that MDD was associated with a specific pattern of overrepresentation of certain order of bacteria (Bacteroidales), as well as specific strains (e.g. *Oscillibacter*), and an underrepresentation of certain families (Lachnospiraceae). Employing a similar methodology, Zheng and colleagues (2016) compared gut microbiota composition between MDD patients and healthy controls and reported significant differences in the relative abundance of phyla Firmicutes, Actinobacteria and Bacteroidetes. Further, they performed faecal transplants from MDD patients to germ-free mice which resulted in depression-like behaviours, while transplants from healthy controls did not. These latter results were also replicated by Kelly et al. (2016). In a gut microbiome analysis study in patients with bipolar disorder, Evans et al. (2017) showed that the fractional representation of *Faecalibacterium* [phylum Firmicutes] was associated with better scores in self-reported measures of depression, anxiety, sleep and mania.

Messaoudi et al. (2011) demonstrated in a double-blind placebo-controlled study that a combination of *Lactobacillus helveticus* and *Bifidobacterium longum* reduced depressive and anxiety symptoms in a non-clinically depressed population. Two other studies, one in healthy volunteers and one in patients with chronic fatigue syndrome, demonstrated that consumption of *Lactobacillus casei* leads to improvements in self-rated mood and clinical measures of depression and anxiety, respectively (Benton et al., 2007; Rao et al., 2009). Other studies that used multispecies probiotic supplementation

have reported a significant reduction in cognitive reactivity to depressive thoughts (Steenbergen et al., 2015) and significant improvement in people suffering from stress and exhaustion (Gruenwald et al., 2002).

Only three studies in clinically depressed individuals have been published to date, two of which administered probiotics as add-on to antidepressants and one as a standalone treatment (key characteristics of the studies are summarised in Table 1). Akkasheh et al. (2016) and Kazemi et al. (2018) administered probiotic supplements to patients with a diagnosis of MDD for 8 weeks and reported a significant decrease of Beck Depression Inventory (BDI) total score in the probiotic group compared to placebo. Romijn et al. (2017) administered probiotics for 8 weeks to medication-free self-referred individuals with an unconfirmed diagnosis of MDD. They found no significant difference between groups after treatment. Taken together, this limited evidence suggests that there may be a beneficial effect of probiotics on depressive symptoms in MDD when administered in addition to antidepressants. However, there are multiple methodological differences in the published studies, which limit the ability to draw clinically meaningful conclusions. Partially this is due to a lack of consensus at present on which bacterial strains and at what doses may be most effective for treating depressive symptoms. This stems from gaps in our understanding of the changes of the composition of the gut microbiota in depression.

Table 1: Summary of the key characteristics of the published RCTs of probiotics in clinical depression.

Author Year	Sample	Sample size	Intervention type	Intervention length	Probiotic strains (CFU)/g and dose	Control arm(s)
Akkasheh et al. 2015	MDD patients aged 20-55	40	Add-on	8 weeks	*L.acidophilus* ($2x10^9$)<br>*L. casei* ($2x10^9$)<br>*B. bifidum* ($2x10^9$)/ g<br>1 capsule daily	placebo
Romijn et al. 2017	Self-referrals with at least moderate depression score; aged >16	79	Primary	8 weeks	*L.helveticus*<br>*B.longum*<br>($\geq 2x10^9$)/g<br>1.5 g sachet daily	placebo
Kazemi et al. 2018	MDD patients aged 18-50	110	Add-on	8 weeks	*L.helveticus*<br>*B.longum*<br>($\geq 2x10^9$)/g<br>5g sachet daily	placebo<br>prebiotic

Adapted from Nikolova et al. 2018 (*under peer review*)

Furthermore, the mechanisms through which probiotics modulate brain function and psychological outcomes are not fully understood. Evidence from animal studies has suggested that the antidepressant action of probiotics is produced via increase of serum levels of (i) tryptophan (Desbonnet et al., 2008); and (ii) brain derived neurotrophic factor (BDNF) (Rios et al., 2017); (iii) decrease of hypothalamus-pituitary-adrenal (HPA) axis activation (Ait-Belgnaoui et al., 2014); and (iv) reduction of pro-inflammatory cytokines (e.g. IL-1β, IL-6, CRP, TNFα) (Cryan and Dinan, 2012).

Few studies to date have employed imaging methods to investigate the impact of gut bacteria on the brain. Bagga et al. (2018) used functional magnetic resonance imaging (fMRI) to investigate the effects of a 4-week treatment with a multi-strain probiotic on brain activity during emotional processing tasks in healthy volunteers. They reported significant alterations in brain areas involved in emotional decision-making and emotional memory, which were correlated with changes in gut microbiota composition. Similarly, Tillisch et al. (2013) observed that a 4-week intake of a fermented milk product containing probiotics affected activity in brain regions involved in emotional processing in healthy women. Additionally, a recent magnetic resonance spectroscopy (MRS) study showed that

Lactobacillus rhamnosus can increase levels of GABA, N-acetyl aspartate and glutamate in the mouse brain (Janik et al., 2016). These mechanisms are yet to be investigated in humans and, more specifically, in patients with MDD.

2.Study Objectives and Design

2.1.Study Objectives

The primary objectives (PO) of this study are:

1. to characterise the gut microbiota in MDD patients (by subjecting the baseline samples of all MDD participants to microbiome analysis);
2. to investigate gut microbiota differences between MDD patients and healthy controls (by comparing the baseline MDD samples with demographically matched healthy volunteer samples);
3. to analyse the changes to the gut microbiota in MDD patients induced by probiotic treatment and their correlation to change in depressive symptoms between baseline and week 8.

The secondary objectives (SO) of this study are:

1. to investigate whether there are changes in blood levels of pro-inflammatory cytokines after 8 weeks of probiotic treatment and whether these mediate the effects of probiotics on mood;
2. to investigate whether there are changes in glutamate and GABA levels in the brain after 8 weeks of probiotic treatment (as measured by MRS);
3. to investigate whether probiotics affect response to emotional faces (in terms of accuracy of recognition and brain activity, as measured by fMRI);

Tertiary objectives (TO):

- to examine whether any changes to the above-mentioned variables (with the exception of neuroimaging) can be observed at 4 weeks after probiotic initiation;
- to check the effectiveness of the blinding manipulation

Table 2 provides a summary of the data that will be used for testing each of the stated objectives.

Table 2: Types of data/samples that will be used for the testing of each stated objective with the corresponding timepoint.

Outcome	Population	Type of data/sample collected	Data collection timepoint
**PO 1**	MDD	Stool	Baseline
**PO 2**	MDD<br>HV	Stool, questionnaire	Baseline
**PO 3**	MDD probiotic<br>MDD placebo	Stool, questionnaire	Baseline & week 8
**SO 1**	MDD probiotic<br>MDD placebo	Stool, blood, questionnaire	Baseline & week 8
**SO 2**	MDD probiotic<br>MDD placebo	Neuroimaging	Baseline & week 8
**SO 3**	MDD probiotic<br>MDD placebo	Neuroimaging, behavioural	Baseline & week 8
**TO 1**	MDD probiotic<br>MDD placebo	Stool, blood, questionnaire, behavioural	Baseline, week 4 & week 8
**TO2**	MDD probiotic<br>MDD placebo	Questionnaire	week 8

2.2.Study Design

This study will employ a double-blind placebo-controlled parallel groups design to investigate the effects of 8 weeks of treatment with probiotics on the gut microbiota, brain activity, levels of glutamate and GABA in the brain, and pro-inflammatory cytokines in patients with MDD.

To investigate the differences in the gut microbiota between MDD patients and healthy individuals, we will also collect stool samples from 25 demographically-matched healthy volunteers and compare these with the MDD stool samples collected at baseline.

Healthy volunteers will not undergo probiotic treatment, therefore, the sections below referring to probiotics, randomisation & blinding and lifestyle guidelines are only relevant to the MDD population.

2.3. Flowchart of the double-blind study

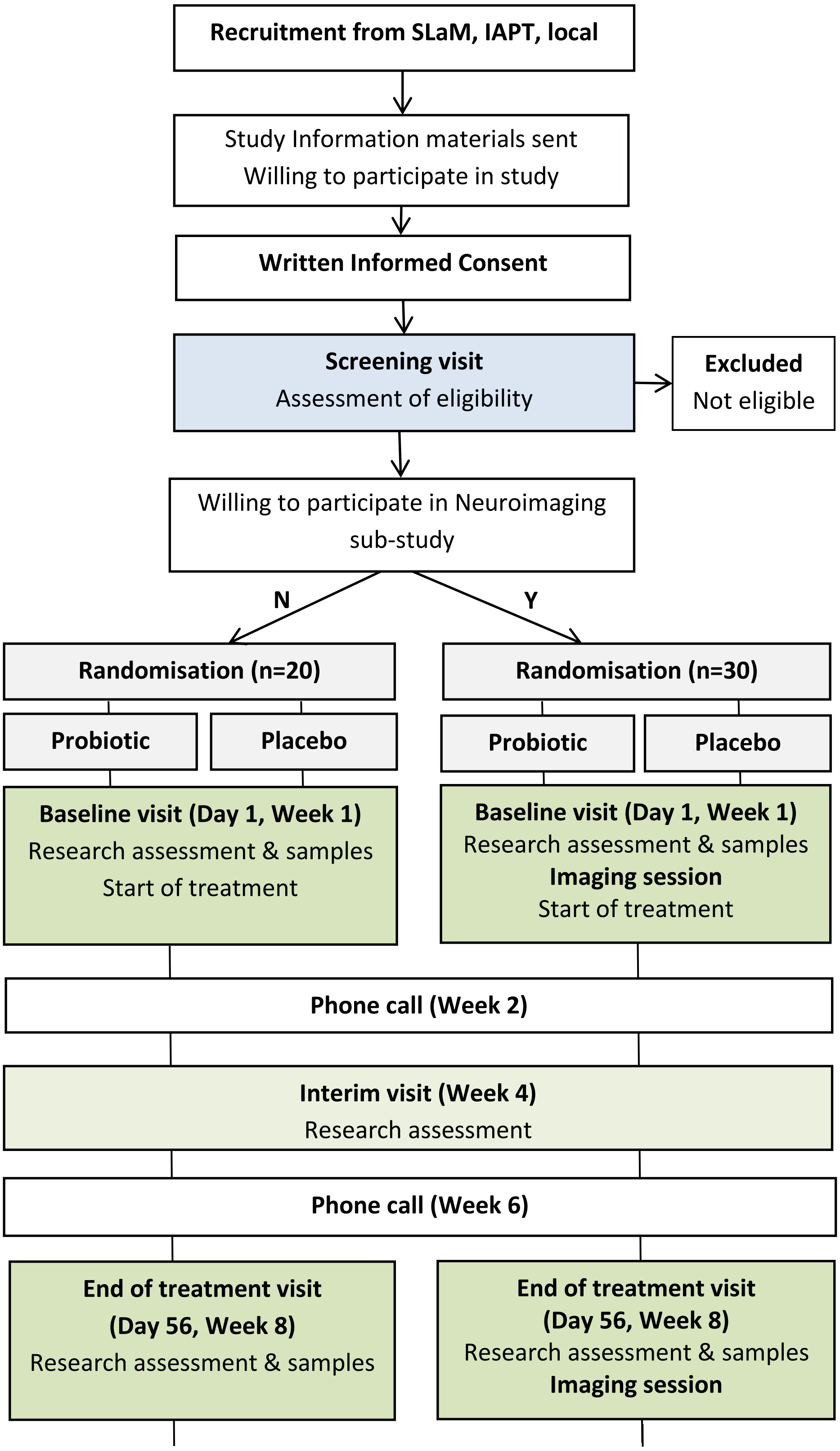

3.Sample Size and Statistics

3.1. Power Calculation and Sample Size

As this is an exploratory study, the data generated in this study will be used to calculate effect sizes to inform sample size calculations for future studies.

At present, it is difficult to perform power analysis for microbiome studies in psychiatric populations because the effect sizes of psychiatric disorders are yet unknown. To our knowledge, only four studies have examined the gut microbiome composition in affective disorders in comparison to healthy controls. The sample sizes in these papers have ranged from n=18 to n=63 per group, with divergent analysis methodologies used (Kelly et al., 2016; Naseribafrouei et al., 2014; Zheng et al., 2016; Jiang et al., 2015). In some of these studies, however, the groups were not matched on key demographic variables (e.g Kelly et al., 2016), known to greatly affect the microbiome.

In keeping with trends in current literature, to test our PO1 we have chosen a sample size of 50 MDD participants. For PO2, we will collect samples from 25 demographically-matched non-depressed healthy participants and perform comparative analyses with the MDD participants. A preliminary calculation performed with G*Power 3.1.9.2, suggests that a group size of 25 participants is powered at 0.8 and alpha level set at 0.05 to detect an effect size of 0.8.

For PO3, the following power calculation was performed: to detect a correlation of 0.6 between change in gut microbiota and change in mood rating scales, a sample size of 17 per group is required. For this study, the sample size of 50 MDD participants will allow for approx. 26% attrition rate at week 8. This was the attrition rate reported at week 8 in the most recent RCT of probiotics for MDD (Kazemi et al., 2018).

There have been no fMRI studies of probiotics in depressed populations, nor any MRS studies of probiotics in human. Therefore, our neuroimaging sub-study (n=30) is exploratory and will generate pilot data that will be used to inform future power analyses.

3.2.Statistical Analysis

Stool samples will be subjected to microbiome analysis. Species diversity at the level of organisational taxonomic units (OTUs) will be investigated with the most up-to-date bioinformatics pipelines at the type of analysis and the significance of any differences between MDD patients and healthy controls will be tested using series of Student's t-tests, corrected for multiple comparisons. Differences in the change of the microbiome of MDD patients following probiotic vs. placebo treatment will also be analysed with Student t-tests. Associations between change in bacterial species levels and change in rating scale scores as well as secondary outcome measures and covariates will be tested using General Linear Model (MANCOVA). Effect sizes will be calculated for all analyses.

4.Study Procedures

4.1.Selection and Withdrawal of Participants

Recruitment

Participants with MDD will be recruited primarily through South London and Maudsley NHS Foundation Trust clinical services, including the 'Consent for Contact c4c' initiative and the Improving Access to Psychological Therapies (IAPT) service. Local KCL/SLaM email circulars and flyers will also be used. Participants will also be recruited through other ethically approved studies where participants

have previously consented to be contacted for other studies (e.g. the Genetic Links to Anxiety and Depression (GLAD) study), by word of mouth, through study adverts on recruitment websites (e.g. Gumtree, www.callforparticipants.com), charity websites and social media. The GLAD study is a research resource comprised of re-contactable participants who have provided online consent and demographic and clinical information via an online questionnaire and has been REC approved (Ref: 18/LO/1218). Healthy volunteers will be recruited via the same means as MDD participants, except for the clinical services.

Inclusion criteria for Healthy Volunteer participants

1. Aged 18-55;
2. No current or historic presence of depression, other psychiatric disorder or substance dependence (confirmed with the Mini International Neuropsychiatric interview (MINI) (Sheehan et al., 1998);
3. No evidence or history of a systemic medical illness;
4. No family history of psychiatric disorder;
5. Non-smokers (assessed by Fagerstrom smoking scale (Fagerström, 1978);
6. Not used probiotic supplements or live yoghurt in the past 2 weeks, nor regular use of a probiotic;
7. Not used antibiotics in the past 12 weeks;
8. No current presence of gastrointestinal (GI) problems (measured with GSRS) or disease, or history of major GI surgery (with the exception of appendectomy);
9. No reported regular/current use of antacids, proton pump inhibitors, laxatives, antidiarrheals;
10. pregnancy or breastfeeding;
11. not currently following a dietary regimen or dietary restrictions unrepresentative of the general population (e.g. fasting or following a specific diet);
12. Capable and willing to provide informed consent;

Inclusion criteria for MDD participants

1. outpatients in a depressive episode according to DSM-5 criteria (confirmed with MINI); who are currently receiving treatment but remain symptomatic (with a Hamilton Depression Rating Scale (HAM-D17) (Hamilton, 1960) score of >13), where the depressive episode cannot be explained by/ or is secondary to another co-morbid medical or psychiatric diagnosis
2. aged 18-55;
3. on a stable treatment regimen of an approved treatment for at least 6 weeks and willing to remain on the same medication and dose throughout the study;
4. non-smokers (assessed by Fagerstrom smoking scale (Fagerström, 1978);
5. capable and willing to provide informed consent;
6. able to attend all study visits and comply with the blood and stool sampling requirements;
7. For participants taking part in the imaging sub-study only: right-handed (assessed by the Edinburgh Handedness Inventory (Caplan and Mendoza, 2011).

Exclusion criteria for MDD participants

1. presence of an eating disorder, bipolar disorder, schizophrenia or psychotic symptoms, personality disorder (assessed with MINI or medical history (self-report));
2. substance dependence in the past year, except for caffeine (assessed with MINI);
3. active suicidal ideation based on MINI and HAM-D17 suicidality item score >2;
4. reported use of probiotic supplements or live yoghurt in the past 2 weeks, or regular use of a probiotic;
5. reported use of antibiotics in the past 12 weeks;

6. history of allergic reaction to any of the components of BioKult;
7. reported history of HIV, cancer or other serious condition at the discretion of the PI;
8. reported current presence of significant GI problems (measured with GSRS) or disease or major GI surgery (with the exception of appendectomy);
9. pregnancy or breastfeeding;
10. patients will also be excluded if they are following a dietary regimen or dietary restrictions unrepresentative of the general population or are planning a major dietary change during the period of the study (e.g. fasting);
11. reported regular/current use of antacids, proton pump inhibitors, laxatives, antidiarrheals;
12. inability or unwillingness to comply with the lifestyle guidelines;
13. For participants taking part in the neuroimaging sub-study only: Contraindications to MRI scanning, including presence of a pacemaker or other metallic implants, claustrophobia and weight >126kg or physical dimensions such that the participant may not fit in the scanner.

Lifestyle Guidelines (MDD participants only)

Participants should refrain from taking any other probiotic supplements or foods known to contain probiotics such as live yogurt, fermented dairy products (e.g. kefir) or sauerkraut throughout the study. Participants will also be asked to notify the study team of any planned prolonged travels, particularly abroad. Study participation may be deferred until after their return, as per per investigator judgment. Participants will also be asked to not make major dietary or physical activity changes during the study. Adherence to their typical diet will be measured via FFQs at baseline and week 8 which will then be compared. Participants taking part in the imaging sub-study will be asked to not consume alcohol the night before the imaging visit.

Withdrawal

Participants have the right to withdraw from the study at any time for any reason. The right to withdraw is clearly explained in the participant information sheet and consent form. The investigator also has the right to withdraw participants from the study in the event of inter-current illness, AEs, protocol or treatment non-compliance or administrative reasons. Treatment non-compliance will be defined as <80% of doses not taken. As an excessive rate of withdrawals can render the study uninterpretable, unnecessary withdrawal of participants should be avoided. Should a participant decide to withdraw from the study, all efforts will be made to report the reason for withdrawal as thoroughly as possible. Should a participant withdraw from the intervention, efforts will be made to continue to obtain follow-up data, with the permission of the patient.

4.2. Randomisation and Blinding

Participants will be randomized into the study after providing informed consent and meeting the study selection criteria. Randomisation service will be provided by the King's Clinical Trials Unit (KCTU). Randomisation will use the web-based service hosted at the KCTU in accordance with a standard operating procedure and held on a secure server. Each participant will be assigned a unique identification number and randomized at the individual level prior to their baseline visit. Each participant will be randomised 1:1 to probiotic or placebo. The study will be double-blind with participants, Investigators and researchers responsible for data collection being unaware of the allocation throughout. To maintain the blinding, the randomisation lists will be communicated directly to pharmacy staff, without the research team's involvement. Unblinding will occur at the end of data collection on approval by the Investigator.

4.3. Intervention

Description

This study will use the probiotic formulation 'BioKult Advanced' by Protexin, which contains the main bacteria strains demonstrated to have a beneficial effect on depressive symptoms in human studies. BioKult contains: *Bacillus subtilis, Bifidobacterium bifidum, Bifidobacterium breve, Bifidobacterium infantis, Bifidobacterium longum, Lactobacillus acidophilus, Lactobacillus delbrueckii ssp. bulgaricus, Lactobacillus casei, Lactobacillus plantarum, Lactobacillus rhamnosus, Lactobacillus helveticus, Lactobacillus salivarius, Lactococcus lactis ssp. lactis, Streptococcus thermophilus*. Each capsule contains minimum 2 billion live microorganisms per capsule ($2x10^9$ CFU/capsule), equivalent to 10 billion live microorganisms per gram ($1x10^{10}$ CFU/gram). BioKult has a two-year shelf life and guaranteed stability and count for the full two years.

Dose & Directions

Patients will take 4 capsules daily for 8 weeks, as two capsules twice per day. There is no requirement for BioKult to be taken at a certain time of day. However, to increase compliance, participants will be asked to take the doses at the same time every day with a meal. Recent research has suggested that ingesting probiotics directly with a meal enables bacteria to be buffered with the acidity of the stomach (https://www.bio-kult.com/faqs).

Packaging and Labelling

Probiotic and placebo capsules will be manufactured by Protexin and packaged into 120 capsule boxes (8 blisters in each). This packaging is suitable for one dispensing episode and gives participants sufficient supply for 4 weeks. The placebo and probiotic capsules will be visually identical and in identical packaging. Annex 13-compliant labels will be provided by Protexin. As pharmacy staff will not be blinded, the study product will be supplied to pharmacy in containers that will be clearly differentiated and then dispensed to researchers and participants in a blinded fashion.

Storage and Dispensing

All study product will be stored at South London and Maudsley Hospital NHS Pharmacy according to manufacturer instructions (in a cool dry place at room temperature up to 25 degrees). Temperature excursions including quarantine procedures will take place as per KHP Clinical trial pharmacy SOP's. South London and Maudsley Hospital NHS Pharmacy will dispense and maintain the blinding codes provided by the KCTU. There will be two dispensing episodes: at baseline and at week 4 visits.

4.4.Concomitant medication/therapy

Participants will be required to be on a stable treatment regimen of an approved pharmacological or psychological treatment (according to the NICE (2009) and British Association of Psychopharmacology (Cleare et al., 2015) guidelines) for at least 6 weeks and willing to remain on the same treatment throughout the study.

4.5.Study Procedures Schedule by Visit

4.5.1 Healthy Volunteer participants

Healthy volunteers will attend only one study visit at the IoPPN campus, KCL, which will last approximately 2 hrs. At this visit they will provide written informed consent (as detailed below in section 4.5.2 'Informed consent procedure'), will complete the MINI, HAM-D, GSRS, food-frequency questionnaire (FFQ) and demographic information questions and, if determined eligible, will be given a stool sample self-collection kit and guided through the instructions on how to correctly collect the sample. They will then be asked to collect the sample at home either on the same day or up to 3 days after the visit (not including weekends) and bring it to the research site on the day of collection. These samples will be collected, handled, stored and analysed following the same procedures used for the MDD samples outlined in the sections below.

4.5.2 MDD participants

Table 3 presents the overall procedures schedule for the double-blind study. Each MDD participant will attend 4 study visits at the IoPPN campus, KCL: screening, baseline, at week 4 and at week 8 of treatment. In addition, remote assessments will be performed over ever 2 weeks. Participants will be reimbursed for their time and travel at each visit.

Table 3: Study procedures schedule

Procedure	Screening	Baseline	Week 2 (Phone)	Week 4	Week 6 (Phone)	Week 8
Administrative						
Informed Consent	x					
Randomisation		x				
Dispensing probiotic/placebo		x		x		
Screening						
Demographics	x					
MINI	x					
General medical history	x					
Depression history + HAM-D17	x					
Depression treatment	x					
Edinburgh Handedness Inventory	x					
Fagerstrom smoking scale	x					
Eligibility Assessment	x					
Biological samples						
Blood sample (TNF-a,IL-1β,IL-6,IL-17,CRP,BDNF)		x		x		x
Stool sample		x		x		x
Assessments						
Weight, BMI	x	x		x		x
Researcher-rated: HAM-D17, CGI, HAM-A		x		x		x
Participant-rated: IDS30-SR, GAD-7, EQ-5D		x		x		x
CTQ		x				
PDQ-20		x		x		x
Emotional Recognition task		x				x
*Neuroimaging**						
fMRI Emotional faces task		x				x
1H-MRS		x				x
Monitoring						
GI symptoms (GSRS)	x	x	x	x	x	x
Adverse Events		x	x	x	x	x
Concomitant Medication		x	x	x	x	x
Lifestyle guidelines adherence		x	x	x	x	x
Capsule count (intervention adherence)				x		x
FFQ		x		x		x
Bristol Stool chart		x		x		x
Success of Blinding check						x
COVID-19 questionnaire		x		x		x

***for a subset of 30 participants only**; MINI - Mini International Neuropsychiatric interview; IDS30-SR- Inventory of Depressive Symptomatology-Self Rated, 30-Item; CGI – Clinical Global Impression scale; GAD – Generalized Anxiety Disorder; HAM-D17- Hamilton Depression Rating Scale 17-item, HAM-A - Hamilton Anxiety Rating Scale; EQ-5D– EuroQol 5 Dimension scale; GSRS-

Gastrointestinal Symptom Rating Scale; CTQ – Childhood Trauma Questionnaire; FFQ- food frequency questionnaire; PDQ-Perceived Deficits Questionnaire;

Informed Consent Procedure

At the screening visit the Principal Investigator, or appropriately trained person delegated by the Principal Investigator as documented in the site delegation log, will obtain written informed consent from each participant prior to any study specific procedures. Viktoriya Nikolova will attend the King's College London Human Tissue Act and Consent training to be appropriately qualified to obtain consent. The Informed Consent procedure will be preceded by an adequate explanation of the aims, methods, anticipated benefits and potential risks of the study. The participant will be given ample time to consider giving their consent for the study. It will be explained to the potential participant that they are free to refuse participation or alternatively withdraw their consent at any point during the study and for any reason. All participants who are actively enrolled on the study will be informed of any updated safety information which may result in significant changes in the risk/benefit analysis and will be re-consented to confirm their wish to continue the study.

Screening visit

The potential participant will be first contacted regarding the study by a researcher from the study team who will send them a copy of the Participant Information Sheet and Consent form (via email or post). The researcher will encourage potential participants to spend as much time as they need to read this information, ask questions about the study and consider whether they wish to participate. If interested in taking part, they will be asked a set of basic pre-screening questions over the phone (such as age and whether they are currently receiving treatment for depression) to determine their suitability for the study before being asked to attend a screening visit. Full written informed consent will be obtained (as detailed below) and an assessment of eligibility will be performed according to the eligibility criteria outlined above. If determined eligible, the participant will be randomized to probiotic or placebo before the baseline visit so that dispensing can take place on the day of the baseline visit.

In addition, eligible participants will be familiarised with the stool sample collection kits and taken through the instructions for collection. They will be given a copy of the instructions and a kit to take home so that they can collect the first sample on the day of the baseline visit prior to arriving at the research site (if possible).

Baseline visit

The baseline visit will take place within 14 days of screening. After re-confirmation of eligibility, the following procedures will be performed:

<u>All participants</u>:

- Self-collection of stool sample;
- blood sample collected by a trained nurse or researcher for inflammatory marker analysis;
- questionnaires: IDS-SR 30 (Rush et al., 2000), HAM-17 (Hamilton, 1960), HAM-A(Hamilton, 1959), EQ-5D (Rabin and de Charro, 2001), GAD-7 (Spitzer et al., 2006), CGI (Guy W., 1976), CTQ (Pennebaker and Susman, 1988), PDQ-20 (Sullivan et al., 1990)
- food frequency questionnaire (FFQ) (Cleghorn et al., 2016) to serve as baseline measure of their typical diet; Bowel movement consistency will be rated using the Bristol Stool Chart (Lewis and Heaton, 1997)

- Adverse Events (AEs) and gastro-intestinal symptoms (GSRS) (Svedlund et al., 1988) monitoring
- Emotional Recognition task
- Weight, BMI

Sub-group of 30 participants (15 per group):

- 1 hr imaging session at the Centre for Neuroimaging Studies (CNS) including a resting state scan, fMRI while performing an emotional faces task and 1H-MRS.

At the end of the baseline visit, all participants will be dispensed probiotic/placebo and instructed to take 2 capsules at the same time every day, directly before a meal. This is in line with manufacturer recommendations and will help ensure compliance.

Phone assessments (week 2, week 6)

A researcher from the team will telephone participants to perform a brief mental state and treatment and lifestyle guidelines compliance check and to collect information on adverse events.

Interim visit (Week 4, day 28 ± 3)

The following procedures will be performed with all participants, as described above: weight, BMI, blood and stool samples, mood and quality of life questionnaires, and AE and gastro-intestinal symptoms monitoring. Lifestyle guidelines adherence check and treatment compliance check (by capsule count) will also be performed. Participants will be dispensed the second batch of probiotic/placebo for the remaining 4 weeks of the study.

End of treatment visit (Week 8, day 56 ± 3)

All participants:
All procedures performed at baseline will be repeated (with the exception of CTQ), as well as treatment compliance (capsule count) and lifestyle guidelines adherence. The success of blinding will be assessed by asking participants 'What do you think you received?: probiotic/ placebo/ can't tell'.

Sub-group of 30 participants (15 per group):

- 1 hr imaging session will be performed as described above.

Emotional Recognition task (computer based)

Biases in processing emotional stimuli have been consistently noted in depression, with depressed patients having a tendency to rate ambiguous expressions as more negative (Bland et al., 2016). This suggests that emotional face recognition may be a sensitive biomarker for depression.

All participants in this study will perform a shortened version of the Emotional Recognition Task (ERT), part of the CANTAB test battery (Cambridge Cognition Ltd), in which they will be shown a series of faces and asked to identify the emotion (happiness, sadness, anger, fear). The total duration of the task is 12min. This shortened version of the task is part of the EMOTICOM battery and has shown good reliability and validity (Bland et al., 2016).

Neuroimaging sub-study

A subset of fifteen participants per group will undergo a one-hour imaging session performed by qualified radiographers using a 3T GE MRI scanner located in the Centre for Neuroimaging Studies, King's College London. 1H-MRS imaging at rest and fMRI imaging while viewing emotional faces at

baseline and at week 8 in order to examine changes in glutamate and GABA neurotransmitter levels and in brain connectivity during emotional activation.

MRI imaging will commence with an initial localizer scan followed by acquisition of structural images including a whole-brain 3D 1mm resolution sagittal T1-weighted scan (ADNI-GO with parallel imaging factor 2), an axial 2D PD and T2-weighted fast spin echo scan, and an axial fast fluid-attenuated inversion recovery scan (total scan time = 10 min). An emotional faces fMRI task (as described below) will then be performed. Baseline 1H-MRS data will then be acquired from the ACC. GABA-edited MR spectra will be acquired using the MEGA-PRESS and methods (Mescher et al., 1998), from a 30×30×30mm volume. An estimation of ACC glutamate (2x2x2cm voxel placed 13mm above the anterior part of the genu of the corpus callosum at 90o to the AC-PC line) will be acquired using HERMES. Unsuppressed water reference spectra will be acquired at the same time from the same voxel locations.

Emotional faces fMRI task

When administered the emotional faces task, depressed patients showed significantly greater subgenual anterior cingulate activation to positively valenced images than controls (Gotlib et al. 2005). Further, multiple antidepressants have been shown to alter emotional faces processing in depressed patients and these changes have been linked to changes in neural activation (Rawlings et al., 2010). Here, we will measure the effect of probiotics on neural activation during the emotional faces task. The emotional faces task uses a well-validated protocol where participants are asked to identify whether faces are male or female whilst being presented faces in blocks of 10 displaying different emotions (Gotlib et al., 2005).

Neuroimaging data analysis

MEGA-PRESS and PRESS spectra will be analyzed using LCModel (Provencher 1993). T1-weighted scans will be segmented into grey matter, white matter and cerebrospinal fluid (CSF) using Statistical Parametric Mapping 8 software to allow correction of water-scaled metabolite values for partial volume CSF contamination. Changes in glutamate and GABA levels pre- and post- treatment will be compared using unpaired t-tests. The change in levels between the probiotic and placebo groups at the end of treatment will be tested for differences using a general linear model.

Emotional faces fMRI task data analysis

The emotional faces fMRI task will be analysed using a general linear model with group, treatment and emotion as factors. Significant interactions will be clarified using planned comparisons between groups for each emotion.

4.6. End of Study Definition

The study is to be considered completed with the last study assessment for the last participant in the study.

4.7. Progression Milestones

While overall this is a mechanistic study, the double-blind placebo-controlled parallel groups study with MDD patients will also serve as a pilot study. The results of this study will be used to inform sample size calculations for a future large efficacy RCT. All procedures and assessments performed as part of this study are expected to be included in the efficacy RCT. The milestones for progression to a full efficacy clinical trial will be as follows:

Target:	Green (Go)	Amber (Amend)	Red (No-Go)
Recruitment feasibility (participants randomised overall, % of target)	85%	70-84%	<70%
Treatment adherence (doses taken, % of total)	90%	66-89%	<66%
Study procedures acceptability and compliance (% of planned data and samples collected at primary endpoint)	85%	66-84%	<66%
Acceptability of stool sample donation (% of eligible participants who provide consent for the study) *	66%	50-65%	<50%

*reasons for refusal to participate will be recorded and analysed

5.SAMPLE HANDLING, ANALYSIS AND LABORATORIES

5.1.Stool samples

Sample handling

The sample handling and collection procedures have been planned in a way to minimise the burden on participants as much as possible, while still maintaining appropriate collection of samples for meaningful analyses. Stool samples will be collected by participants using the self-collection kits (Alpha Laboratories, Eastleigh, UK) at the specified timepoints. Stool samples will be collected either at home or during the visit on site (for MDD participants only), kept in the provided container and frozen at −80°C on site within 24 hours of sample collection. Participants will not be required to freeze the samples at home. Previous studies have shown that the effects of short-term storage conditions on the diversity and structure of bacterial communities in stool samples are negligible, however, the number of freeze-thaw cycles does have an impact (Goodrich et al., 2014). To minimize the number of freeze-thaw cycles, all samples will be aliquoted into four cryovials, containing approx. 200mg of stool each before freezing at−80°C. Samples will be labelled with study ID, researcher name, participant study ID and sample type and stored in an HTA designated freezer at the research site. Any surplus material will be discarded according to KCL HTA SOPs for 'Disposal of waste human material' and 'Disposal of surplus and unusable human material' and a record maintained. The sample handling procedure is described in further detail in SOP 01 'Stool Collection, Handling, Storage & Disposal'. Samples will be transported on dry ice in bulk batches to Quadram Institute Bioscience (QIB) by certified courier following the KCL HTA SOP for 'Transfer of human material to other establishments for research purposes'. The PI will ensure that Viktoriya Nikolova has the appropriate and up-to date training for the handling of samples and that a third-party agreement is in place with the courier company prior to any transportation of samples.

Analysis of samples

Samples will be subjected to microbiome analysis, which will be carried out at QIB and led by Dr Lindsay Hall and her team comprised of expert microbiome technicians and bioinformaticians. Viktoriya Nikolova will also perform these analyses following the relevant training and under the supervision of Dr Hall. An agreement between KCL and QIB will be signed prior to any transportation of samples or analyses taking place.

The microbiome of each group will be described in terms of individual taxon abundance and overall measures of diversity. The microbiome analysis will be performed using high-throughput sequencing (e.g. Illumina Mi/HiSeq) following standardised, validated and quality-controlled protocols from Dr Hall's lab, including: 16SrRNA-based sequencing (genus level profiles); shotgun metagenomics; transcriptomics for species-level taxonomic identification; quantitative polymerase chain reaction (PCR) for probiotic colonisation. Bioinformatics pipelines are constantly being improved and updated,

thus the most appropriate ones available at time of analysis will be used. An example can be found in the recently published paper by Alcon-Giner et al. (Alcon-Giner et al., 2017) from Dr Hall's lab. Downstream statistical analyses will be performed using different software and packages e.g. SPSS, Prism, R. For the comparative analyses of differences in OTU level data between healthy controls and MDD patients, series of Student t-tests will be performed with multiple comparison correction. The same analysis will be used to examine the differences in change in the microbiome (e.g. differences in changes of organisational taxonomic units (OTU) level data) between probiotic and placebo groups post-treatment. To examine associations between the species diversity and OTU levels of the microbiome and questionnaire data, behavioural data, inflammatory marker data, and imaging data, Generalized Linear Model (MANCOVA) will be used.

5.2. Blood samples handling & analysis

Blood samples will be collected at baseline, week 4 and week 8 visits from MDD participants only. Blood samples will be collected by a phlebotomy trained nurse or member of the research team and labelled with a unique barcode that includes the participant number and study identifier. The samples will then be transported across Institute of Psychiatry, Psychology and Neuroscience campus to the laboratory facility that will perform the analyses. The inflammatory marker analyses (TNF-a, IL-1β, IL-6, IL-17, CRP, BDNF) will be performed by the laboratory of the NIHR BRC BioResource and the results returned to the study team via email. Samples will be stored and processed in accordance with strict health and safety guidelines and under the requirements of the HTA. King's College London holds an HTA license, number: 12293, which BioResource is covered by. The samples will only be used for the purposes of this study and then discarded. These will not become part of BioResource's collection.

6.ASSESSMENT OF SAFETY

Data on Adverse Events will be collected with open ended questions and recorded systematically. Any reported events will be reviewed by the Investigator.

Even though probiotics are classed as food supplements and not medicinal products, the standard definitions of Adverse events/ Serious Adverse Events for medicinal products will be used, as follows:

Adverse Events (AE) - any untoward medical occurrence in a participant administered a medicinal product. An adverse event can be any unfavourable and unintended sign (including an abnormal finding), symptom, or disease temporally associated with the use of a medicinal product, whether or not related to that medicinal product or to the participants participation in the protocol. This includes any occurrence that is new in onset or aggravated in severity from the baseline condition, or abnormal results of any diagnostic procedures that are conducted within clinical practice. Any adverse event that occurs between the participant consenting to the clinical research through to their last visit (week 8) will be recorded. Investigators will assess whether the adverse event may be related to the participant's participation in the study and will also assess the severity of the event. All documented adverse events reported after participants signs the consent form will be included in the analysis.

Serious Adverse Events (SAE) - any untoward medical occurrence that: results in death, is life-threatening, requires inpatient hospitalization, results in persistent or significant disability/incapacity, is a congenital anomaly/birth defect; and any untoward medical occurrence considered medically significant.

All SAE's will be reported directly by the Investigator within 24 hours of them becoming aware to the sponsor, King's College London, using a SAE Report Form. These will also be reported to Protexin subsequently. All serious adverse events that have not resolved by the end of the study, or that have not resolved upon discontinuation of the subject's participation in the study, will be followed until:

the event resolves, the event stabilizes, the event returns to baseline (if a baseline value is available), the event can be attributed to agents other than the study drug or to factors unrelated to study conduct, when it becomes unlikely that any additional information can be obtained. All follow-up information for SAE's that have not resolved by the end of the study or by the time of participant withdrawal will be reported to the sponsor, King's College London.

7.IMPACT OF COVID-19

The COVID-19 Pandemic occurred during the course of this study's recruitment and data collection phases. To account for the impact of the Pandemic on study outcomes we will introduce a brief questionnaire asking participants whether and when they have: 1) had a prior diagnosis of COVID-19 infection; 2) been hospitalised as a result (as an indication of severity); 3) received any vaccination doses against COVID-19; and 4) experienced any long-lasting symptoms ('long COVID'). This information will be used to assess whether COVID-19 illness or vaccination history may have an impact on study results (i.e., as a potential confounding variable). Specifically, it is important to ensure that any differences between groups (probiotic vs placebo) cannot be attributed to differences in COVID-19 illness/vaccination history.

8.ETHICS AND REGULATORY APPROVALS

The Chief Investigator will ensure that REC Favourable Opinion, HRA approval, and SLaM NHS Trust Confirmation of Capacity and Capability are in place before recruitment for the study begins. This protocol and other study documents have been submitted for review to London - Surrey Research Ethics Committee (REC reference: 19/LO/0761). This study has received MHRA Notification of CTA exemption.

9.DATA HANDLING

The Chief Investigator will act as custodian for the study data. All participant data will be anonymised and stored in password-protected files. Paper forms of participant data will be stored securely on site in locked filing cabinets. Information with regards to study subjects will be kept confidential and managed in accordance with the Data Protection Act, NHS Caldicott Guardian, The Research Governance Framework for Health and Social Care and Research Ethics Committee Approval. Data will be stored for 10 years after study completion and will be archived according to King's College London policy.

During study visits data will be collected on paper Case Report Forms (CRFs) and then transferred to a secure web-based electronic database (REDCap). All electronic data entries will be regularly verified with source data.

10.DISSEMINATION

The results from this study will be written up and published in academic journals and presented at academic conferences. This study also forms a PhD project being conducted by one of the co-investigators at the IoPPN. This project will therefore be written up as a thesis which will be made publicly available through the IoPPN website.

11.FINANCE

This study is funded by the Medical Research Council Doctoral Training Partnership Industrial CASE (iCASE) Studentship programme. The iCASE studentships are part-funded by Research Councils together with an industrial partner, in this case Protexin PLC. Protexin will provide the probiotic and placebo for this study at no cost.

12.REFERENCES

Ait-Belgnaoui A, Colom A, Braniste V, et al. (2014) Probiotic gut effect prevents the chronic psychological stress-induced brain activity abnormality in mice. *Neurogastroenterol Motil* 26: 510-520.

Akkasheh G, Kashani-Poor Z, Tajabadi-Ebrahimi M, et al. (2016) Clinical and metabolic response to probiotic administration in patients with major depressive disorder: A randomized, double-blind, placebo-controlled trial. *Nutrition* 32: 315-320.

Alcon-Giner C, Caim S, Mitra S, et al. (2017) Optimisation of 16S rRNA gut microbiota profiling of extremely low birth weight infants. *BMC Genomics* 18: 841.

Bagga D, Reichert JL, Koschutnig K, et al. (2018) Probiotics drive gut microbiome triggering emotional brain signatures. *Gut Microbes*: 1-11.

Barseghyan K, Alchujyan NK, Aghababova A, et al. (2013) Chronic stress-induced depression-like behavior of rats accompanied by microbial translocation the blood-brain barrier and persistent activation the inducible nitric oxide synthase in mitochondria of cortico-limbic brain. *European Chemical Bulletin* 2: 373-382.

Benton D, Williams C and Brown A. (2007) Impact of consuming a milk drink containing a probiotic on mood and cognition. *Eur J Clin Nutr* 61: 355-361.

Bercik P, Denou E, Collins J, et al. (2011) The intestinal microbiota affect central levels of brain-derived neurotropic factor and behavior in mice. *Gastroenterology* 141: 599-609, 609.e591-593.

Bland AR, Roiser JP, Mehta MA, et al. (2016) EMOTICOM: A Neuropsychological Test Battery to Evaluate Emotion, Motivation, Impulsivity, and Social Cognition. *Front Behav Neurosci* 10: 25.

Caplan B and Mendoza JE. (2011) Edinburgh Handedness Inventory. In: Kreutzer JS, DeLuca J and Caplan B (eds) *Encyclopedia of Clinical Neuropsychology.* New York, NY: Springer New York, 928-928.

Cleare A, Pariante CM, Young AH, et al. (2015) Evidence-based guidelines for treating depressive disorders with antidepressants: A revision of the 2008 British Association for Psychopharmacology guidelines. *J Psychopharmacol* 29: 459-525.

Cleghorn CL, Harrison RA, Ransley JK, et al. (2016) Can a dietary quality score derived from a short-form FFQ assess dietary quality in UK adult population surveys? *Public Health Nutrition* 19: 2915-2923.

Cryan JF and Dinan TG. (2012) Mind-altering microorganisms: the impact of the gut microbiota on brain and behaviour. *Nat Rev Neurosci* 13: 701-712.

Desbonnet L, Garrett L, Clarke G, et al. (2008) The probiotic Bifidobacteria infantis: An assessment of potential antidepressant properties in the rat. Netherlands: Elsevier Science, 164-174.

Evans SJ, Bassis CM, Hein R, et al. (2017) The gut microbiome composition associates with bipolar disorder and illness severity. *Journal of Psychiatric Research* 87: 23-29.

Fagerström K-O. (1978) Measuring degree of physical dependence to tobacco smoking with reference to individualization of treatment. *Addictive Behaviors* 3: 235-241.

Fava M. (2003) Diagnosis and definition of treatment-resistant depression. *Biol Psychiatry* 53: 649-659.

Gareau MG, Wine E, Rodrigues DM, et al. (2011) Bacterial infection causes stress-induced memory dysfunction in mice. *Gut* 60: 307-317.

GBD. (2016) Global, regional, and national incidence, prevalence, and years lived with disability for 310 diseases and injuries, 1990-2015: a systematic analysis for the Global Burden of Disease Study 2015. *Lancet* 388: 1545-1602.

Goodrich JK, Di Rienzi SC, Poole AC, et al. (2014) Conducting a microbiome study. *Cell* 158: 250-262.

Gotlib IH, Sivers H, Gabrieli JD, et al. (2005) Subgenual anterior cingulate activation to valenced emotional stimuli in major depression. *Neuroreport* 16: 1731-1734.

Greenberg PE, Fournier AA, Sisitsky T, et al. (2015) The economic burden of adults with major depressive disorder in the United States (2005 and 2010). *J Clin Psychiatry* 76: 155-162.

Gruenwald J, Graubaum HJ and Harde A. (2002) Effect of a probiotic multivitamin compound on stress and exhaustion. *Adv Ther* 19: 141-150.

Guy W. (1976) *ECDEU Assessment Manual for Psychopharmacology, Revised.* : US Department of Health, Education, and Welfare Publication (ADM) Rockville, MD: National Institute of Mental Health.

Hamilton M. (1959) The assessment of anxiety states by rating. *Br J Med Psychol* 32: 50-55.

Hamilton M. (1960) A RATING SCALE FOR DEPRESSION. *Journal of Neurology, Neurosurgery & Psychiatry* 23: 56-62.

Janik R, Thomason LAM, Stanisz AM, et al. (2016) Magnetic resonance spectroscopy reveals oral Lactobacillus promotion of increases in brain GABA, N-acetyl aspartate and glutamate. *Neuroimage* 125: 988-995.

Jiang H, Ling Z, Zhang Y, et al. (2015) Altered fecal microbiota composition in patients with major depressive disorder. *Brain Behav Immun* 48: 186-194.

Kazemi A, Noorbala AA, Azam K, et al. (2018) Effect of probiotic and prebiotic vs placebo on psychological outcomes in patients with major depressive disorder: A randomized clinical trial. *Clin Nutr*.

Kelly JR, Borre Y, O' Brien C, et al. (2016) Transferring the blues: Depression-associated gut microbiota induces neurobehavioural changes in the rat. *Journal of Psychiatric Research* 82: 109-118.

Lewis SJ and Heaton KW. (1997) Stool Form Scale as a Useful Guide to Intestinal Transit Time. *Scand J Gastroenterol* 32: 920-924.

McCrone P, Dhanasiri S, Patel A, et al. (2008) Paying the Price the cost of mental health care England 2026 King's Fund.

Mescher M, Merkle H, Kirsch J, et al. (1998) Simultaneous in vivo spectral editing and water suppression. *NMR in Biomedicine* 11: 266-272.

Messaoudi M, Lalonde R, Violle N, et al. (2011) Assessment of psychotropic-like properties of a probiotic formulation (Lactobacillus helveticus R0052 and Bifidobacterium longum R0175) in rats and human subjects. *Br J Nutr* 105: 755-764.

Naseribafrouei A, Hestad K, Avershina E, et al. (2014) Correlation between the human fecal microbiota and depression. *Neurogastroenterol Motil* 26: 1155-1162.

NICE. (2009) *Depression in adults: recognition and management*. Available at: https://www.nice.org.uk/guidance/cg90.

Otte C, Gold SM, Penninx BW, et al. (2016) Major depressive disorder. *Nat Rev Dis Primers* 2: 16065.

Pennebaker JW and Susman JR. (1988) Disclosure of traumas and psychosomatic processes. *Soc Sci Med* 26: 327-332.

Prince M, Patel V, Saxena S, et al. (2007) No health without mental health. *Lancet* 370: 859-877.

Rabin R and de Charro F. (2001) EQ-5D: a measure of health status from the EuroQol Group. *Ann Med* 33: 337-343.

Rao AV, Bested AC, Beaulne TM, et al. (2009) A randomized, double-blind, placebo-controlled pilot study of a probiotic in emotional symptoms of chronic fatigue syndrome. *Gut Pathog* 1: 6.

Rawlings NB, Norbury R, Cowen PJ, et al. (2010) A single dose of mirtazapine modulates neural responses to emotional faces in healthy people. *Psychopharmacology (Berl)* 212: 625-634.

Rios AC, Maurya PK, Pedrini M, et al. (2017) Microbiota abnormalities and the therapeutic potential of probiotics in the treatment of mood disorders. *Rev Neurosci* 28: 739-749.

Romijn AR, Rucklidge JJ, Kuijer RG, et al. (2017) A double-blind, randomized, placebo-controlled trial of Lactobacillus helveticus and Bifidobacterium longum for the symptoms of depression. *Australian and New Zealand Journal of Psychiatry* 51: 810-821.

Rush AJ, Carmody T and Reimitz P-E. (2000) The Inventory of Depressive Symptomatology (IDS): Clinician (IDS-C) and Self-Report (IDS-SR) ratings of depressive symptoms. *International Journal of Methods in Psychiatric Research* 9: 45-59.

Sheehan DV, Lecrubier Y, Sheehan KH, et al. (1998) The Mini-International Neuropsychiatric Interview (M.I.N.I.): the development and validation of a structured diagnostic psychiatric interview for DSM-IV and ICD-10. *J Clin Psychiatry* 59 Suppl 20: 22-33;quiz 34-57.

Spitzer RL, Kroenke K, Williams JB, et al. (2006) A brief measure for assessing generalized anxiety disorder: the GAD-7. *Arch Intern Med* 166: 1092-1097.

Steenbergen L, Sellaro R, van Hemert S, et al. (2015) A randomized controlled trial to test the effect of multispecies probiotics on cognitive reactivity to sad mood. *Brain Behav Immun* 48: 258-264.

Sullivan M, Edgley K and Dehoux E. (1990) *A survey of multiple sclerosis: I. Perceived cognitive problems and compensatory strategy use*.

Svedlund J, Sjodin I and Dotevall G. (1988) GSRS--a clinical rating scale for gastrointestinal symptoms in patients with irritable bowel syndrome and peptic ulcer disease. *Dig Dis Sci* 33: 129-134.

Tillisch K, Labus J, Kilpatrick L, et al. (2013) Consumption of fermented milk product with probiotic modulates brain activity. *Gastroenterology* 144: 1394-1401, 1401.e1391-1394.

Zheng P, Zeng B, Zhou C, et al. (2016) Gut microbiome remodeling induces depressive-like behaviors through a pathway mediated by the host's metabolism. *Mol Psychiatry* 21: 786-796.
