# Supplementary material for: Acceptability, Tolerability, and Estimates of Putative Treatment Effects of Probiotics as Adjunctive Treatment in Patients With Depression: A Randomized Clinical Trial
Source: JAMA Psychiatry. 2023 Jun 14;80(8):842–7. doi: 10.1001/jamapsychiatry.2023.1817 (PMC10267847; doi:10.1001/jamapsychiatry.2023.1817)
Supplement: Supplement 2. — eMethods. eFigure 1. CONSORT Diagram of Participant Flow for the PROMEX Study eFigure 2. GI Symptoms (GSRS9) in the Probiotic (Green) and Placebo (Blue) Groups Through the Course of Treatment eFigure 3. Trajectories of Depressive and Anxiety Symptoms in the Probiotic (Green) and Placebo (Blue) Groups Through the Course of the Study eTable 1. Success of Blinding Check eTable 2. Adverse Reactions Experienced by Participants in Either Group eTable 3. Sensitivity Analysis by Race eTable 4. Sensitivity Analysis by Non-SSRI Use eTable 5. Estimates of Treatment Effect on Depressive and Anxiety Symptoms in PP Dataset eTable 6. IDS Items Included in the Rush (1966) Anxiety/Arousal Subscale eReferences. [file jamapsychiatry-e231817-s002.pdf]

Supplemental Online Content

Nikolova VL, Cleare AJ, Young AH, Stone JM. Acceptability, tolerability, and estimates of putative treatment effects of probiotics as adjunctive treatment in patients with depression: a randomized clinical trial. *JAMA Psychiatry*. Published online June 14, 2023. doi:10.1001/jamapsychiatry.2023.1817

eMethods.

eFigure 1. CONSORT Diagram of Participant Flow for the PROMEX Study

eFigure 2. GI Symptoms (GSRS9) in the Probiotic (Green) and Placebo (Blue) Groups Through the Course of Treatment

eFigure 3. Trajectories of Depressive and Anxiety Symptoms in the Probiotic (Green) and Placebo (Blue) Groups Through the Course of the Study

eTable 1. Success of Blinding Check

eTable 2. Adverse Reactions Experienced by Participants in Either Group

eTable 3. Sensitivity Analysis by Race

eTable 4. Sensitivity Analysis by Non-SSRI Use

eTable 5. Estimates of Treatment Effect on Depressive and Anxiety Symptoms in PP Dataset

eTable 6. IDS Items Included in the Rush (1966) Anxiety/Arousal Subscale

eReferences.

This supplementary material has been provided by the authors to give readers additional information about their work.

© 2023 Nikolova VL et al. *JAMA Psychiatry*.

eMethods.

Participants

Outpatients with MDD, according to the Diagnostic and Statistical Manual of Mental Disorders, 5th edition (DSM-5)[1], were recruited from secondary care services and local advertisement in London, UK. Data collection took place between September 2019 and June 2022 with a 15-month interruption of activities due to COVID-19 restrictions.

Intervention

BioKult Advanced® (ADM Protexin, UK) contains 14 strains at a dose of $2x10^9$ CFU per capsule: *Bacillus subtilis PXN®21, Bifidobacterium bifidum PXN®23, Bifidobacterium breve PXN®25, Bifidobacterium infantis PXN®27, Bifidobacterium longum PXN®30, Lactobacillus acidophilus PXN®35, Lactobacillus delbrueckii ssp. bulgaricus PXN®39, Lactobacillus casei PXN®37, Lactobacillus plantarum PXN®47, Lactobacillus rhamnosus PXN®54, Lactobacillus helveticus PXN®45, Lactobacillus salivarius PXN®57, Lactococcus lactis PXN®63* and *Streptococcus thermophilus PXN®66*. This supplement was selected as it contains those bacterial species demonstrated to have a beneficial effect on depressive or anxiety symptoms in pre-clinical and clinical studies of depression (e.g., *L. casei, L. acidophilus, L. helveticus, B. longum, B. infantis, B. bifidum, B. breve, L. bulgaricus, L. rhamnosus, L. salivarius, Lactococcus lactis and Streptococcus thermophilus* have all been used in clinical trials showing positive effects on depressive symptoms[2,3]; extensive species-level pre-clinical summary is available here[4]). This supplement has now also been shown to improve mood in people with self-reported moderate depression[5] and improve mental health parameters in patients with multiple sclerosis[6]. Further, multistrain (compared to single-strain) formulations have shown higher potency in humans and are suggested to exhibit synergistic effects with an expanded benefit on host physiology[4]. Practical advantages were also considered in the selection of the study product. Unlike many others, this product does not need to be stored in the fridge, which was considered beneficial for adherence. Additionally, BioKult Advanced has guaranteed stability of the bacterial count for 2 years and has demonstrated good ability to colonise the GI tract [7,8]. Placebo capsules were identical in appearance and packaging but did not contain any live bacteria. Participants were instructed to take 4 capsules daily with food.

Randomisation, allocation concealment and blinding

Participants were randomized (1:1) to probiotic or placebo through an online system provided by the King's Clinical Trials Unit by a blinded investigator using block randomisation with varying block sizes of 2 and 4. Participants, investigators and lab staff responsible for data collection and sample analysis were unaware of the allocation. Only pharmacy staff received unblinded notifications from the randomisation system and removed identifiable labels from product boxes before dispensing, thus maintaining allocation concealment. Probiotic and placebo boxes, blister packs and capsules were otherwise identical, including serial numbers.

Blinding manipulation success

Success of blinding was assessed by asking participants to guess their allocation at the end of the study. They were presented with three options: probiotic/placebo/don't know and encouraged to make a selection if their first answer was 'don't know', to ensure the credibility of the answer. Responses were then compared between groups (chi-square).

© 2023 Nikolova VL et al. *JAMA Psychiatry*.

Outcome measures

The primary outcome of interest for a future efficacy RCT was change in depressive scores at week 8, measured with HAMD-17 and IDS-SR. One clinician-rated and one patient-rated measure were chosen, as there can be considerable discrepancies between the two types of assessment. Further, the IDS is a detailed measure that allows for the examination of specific depression subtypes and presentations. Other outcome measures included a clinician-rated (HAMA) and a patient-rated (GAD) anxiety scale and a measure of overall clinical status (CGI). These scales are among the most widely used in clinical trials in depression, with demonstrated reliability and validity. Adverse event information was collected through open-ended questions and duration, severity, relatedness, and outcome were recorded. Due to the nature of the intervention, gastrointestinal events were specifically assessed, with the Gastrointestinal Symptom Rating Scale (GSRS)[9]. Data were collected by trained and experienced researchers and total scores calculated following author scoring instructions. No outcome measure data were excluded from analyses.

Statistical analyses

Baseline comparisons were performed using independent samples t-tests, Mann-Whitney U or chi-square tests, depending on variable scale and distribution. The results of these comparisons (p values) were not reported to meet journal requirements of reporting of baseline characteristics in randomised clinical trials. Estimates of treatment efficacy were calculated on both the intent-to-treat (ITT: defined as every participant who took at least one dose of treatment) and per-protocol principles (PP: every participant who completed the treatment and study procedures per protocol) and were aimed at estimating the effect size of the between-group mean difference. To deal with missing outcome data, we used maximum likelihood (ML) approach linear mixed models (LMMRM) under the missing at random (MAR) assumption with the outcomes as the dependent variables and treatment group (probiotic, placebo), time (baseline, week 4, week 8) and time*group interaction as the fixed terms. A random term for participant was also included to account for correlations between the repeated measures. To assess whether the MAR assumption was met (despite the low rate of missingness in the outcome variables, i.e., <10%), we sought baseline predictors of missingness. To that end, logistic regressions with a binary indicator of missingness for each outcome measure were performed. No predictors of missingness were identified, thus maintaining the MAR assumption. For HAMA, sqrt-transformed values were use due to a significant skew of the data and residuals (Shapiro-Wilk<0.05).

Standardised effect sizes (SES) were calculated by dividing the group mean difference by the pooled standard deviation of the relevant outcome at baseline and applying a small sample size correction. The magnitude of effect was interpreted according to convention as small (0.2-0.49), moderate (0.5-0.79) or large (≥0.8).

To evaluate the effect of potential confounders, analyses were repeated with BMI, age, weight, GI complaints (GSRS), alcohol intake and dietary parameters (FFQ) as covariates, once for each covariate. To evaluate the effect of antidepressant medication type and ethnicity imbalance between groups at baseline, sensitivity analyses were performed excluding participants not on an SSRI and those identifying as Asian, non-Chinese.

For CGI-Severity, as an ordinal outcome, generalised linear model (ordinal logistic) was performed as directed by Heck et al. (2012) [10].

Analyses were performed in SPSS (v.28, New York, USA) with significance level set at 0.05.

© 2023 Nikolova VL et al. *JAMA Psychiatry*.

eFigure 1. CONSORT Diagram of Participant Flow for the PROMEX Study

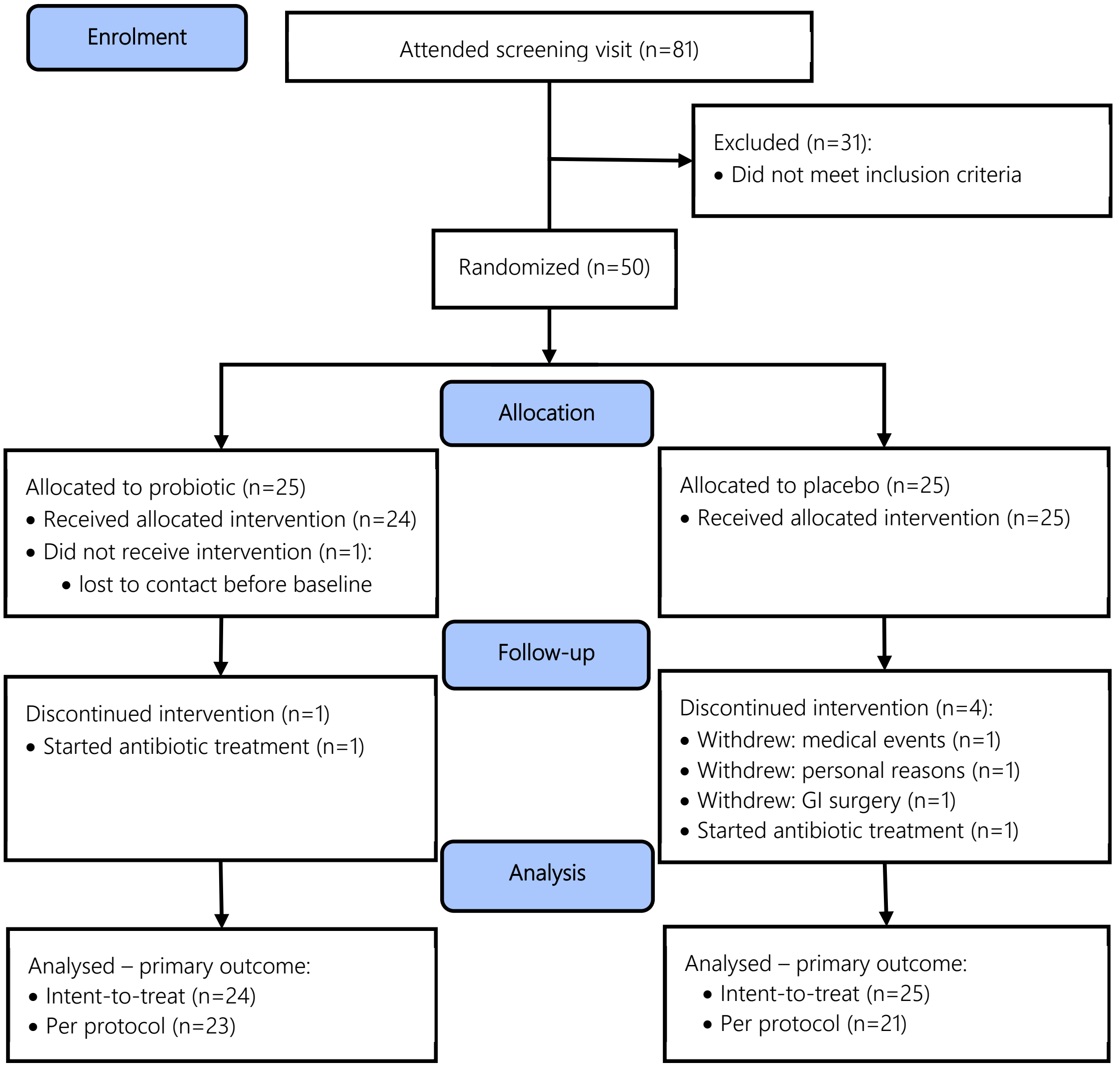

© 2023 Nikolova VL et al. *JAMA Psychiatry*.

eFigure 2. GI Symptoms (GSRS9) in the Probiotic (Green) and Placebo (Blue) Groups Through the Course of Treatment

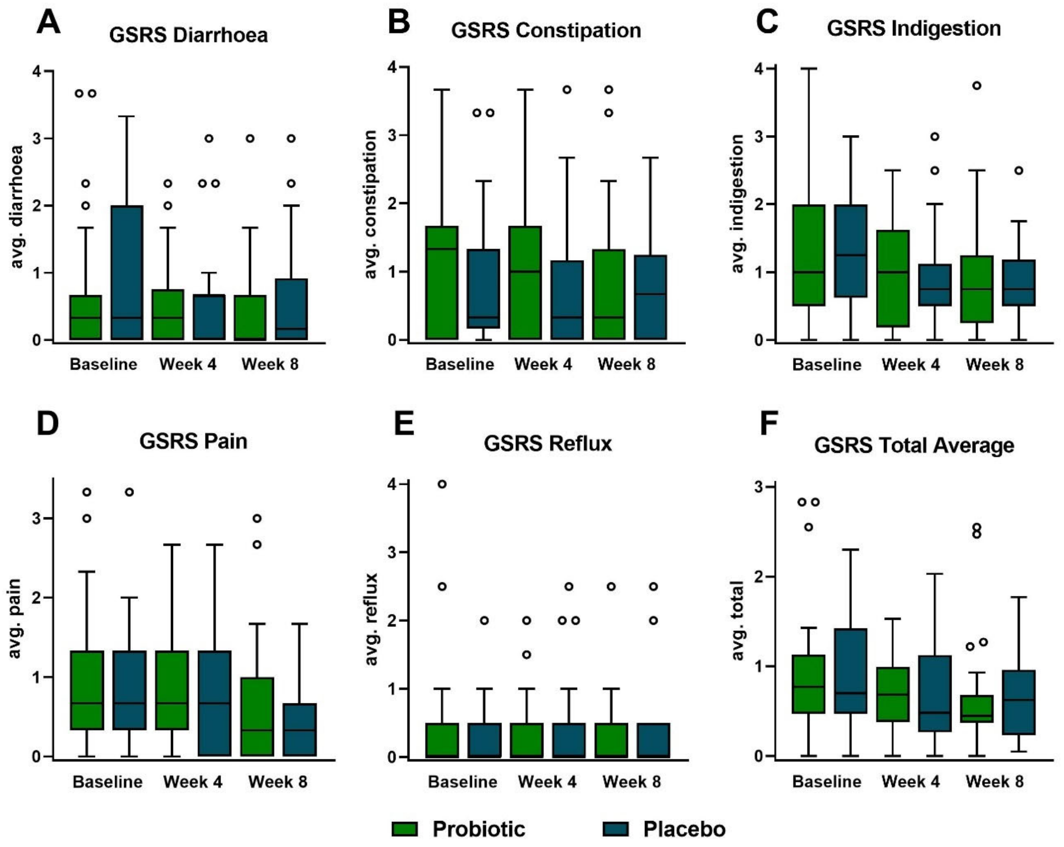

A. Diarrhoea, B. Constipation, C. Indigestion, D. Pain, E. Reflux, F. Average total complaints score. Data are presented as boxplots of untransformed values and Median [IQR], where upper and lower hinges indicate the first and third quartiles and the upper and lower whisker indicate the largest and lowest value, respectively, no further than 1.5 times IQR from the hinge. Data beyond 1.5 times IQR are plotted individually. Per protocol dataset (n=44).

© 2023 Nikolova VL et al. *JAMA Psychiatry*.

eFigure 3. Trajectories of Depressive and Anxiety Symptoms in the Probiotic (Green) and Placebo (Blue) Groups Through the Course of the Study

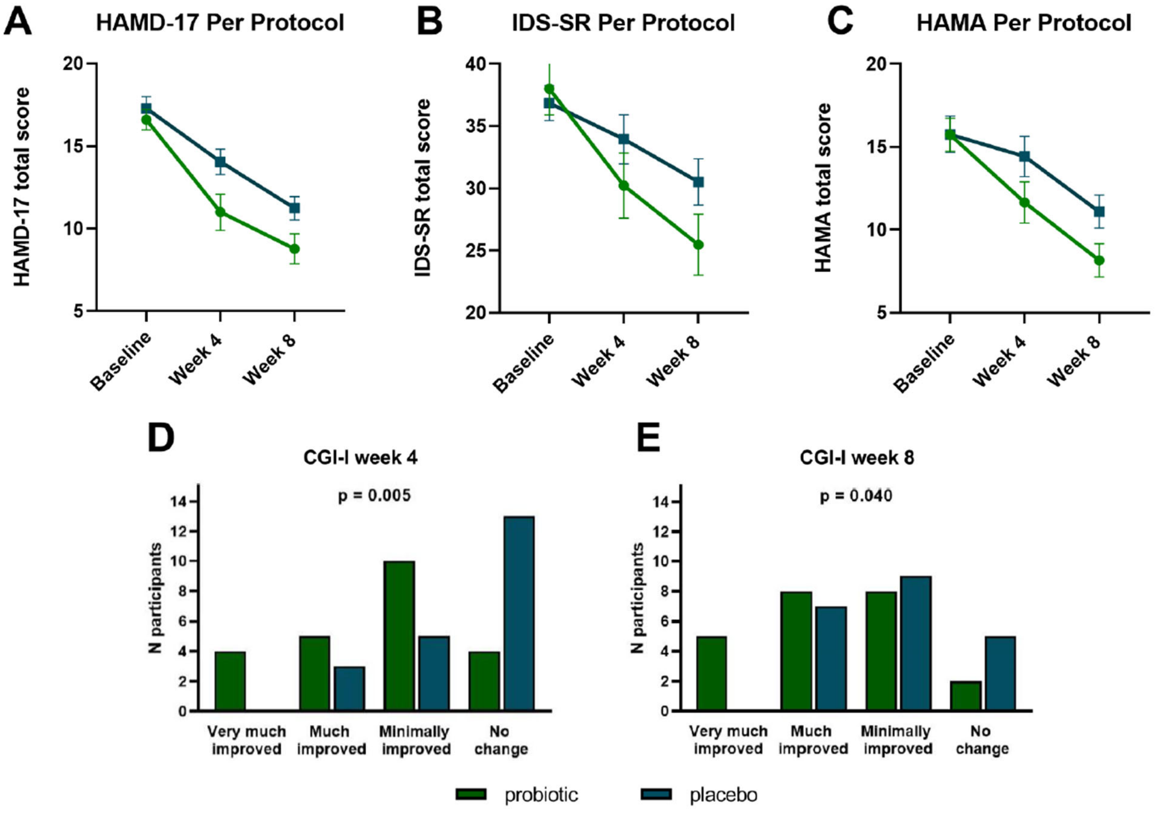

Data from the per protocol dataset (n=44). A-C data are presented as M±SE. D-E p values from Chi square likelihood ratio tests.

© 2023 Nikolova VL et al. *JAMA Psychiatry*.

eTable 1. Success of Blinding Check

**Allocation guess**	**Probiotic group (n=24)**	**Placebo group (n=22)**	**Test statistic**
Placebo n (%)	5 (20.8)	5 (22.7)	
Probiotic n (%)	8 (33.3)	6 (27.3)	
Don't know n (%)	11 (45.8)	11 (50.0)	
Correct n (%)	8 (33.3)	5 (22.7)	$\chi^2(1, 46) = 0.64$, $p = 0.43$

© 2023 Nikolova VL et al. *JAMA Psychiatry*.

eTable 2. Adverse Reactions Experienced by Participants in Either Group

Adverse Event*	Probiotic group (n=24)	Placebo group (n=25)
nausea n (%)	4 (16.7)	0 (0.0)
diarrhoea n (%)	1 (4.2)	1 (4.0)
indigestion n (%)	3 (12.5)	0 (0.0)
bloating n (%)	1 (4.2)	0 (0.0)
constipation n (%)	2 (8.3)	1 (4.0)
acid reflux n (%)	0 (0.0)	1 (4.0)
heartburn n (%)	1 (4.2)	1 (4.0)
stomach ache n (%)	0 (0.0)	1 (4.0)
bloody stool n (%)	1 (4.2)	0 (0.0)
burping n (%)	1 (4.2)	0 (0.0)

* Values indicate number of participants reporting the event

© 2023 Nikolova VL et al. *JAMA Psychiatry*.

eTable 3. Sensitivity Analysis by Race

Estimates of treatment effect on depressive and anxiety symptoms after the removal of all participants identifying as Asian (non-Chinese; n=7), who were randomly allocated to the probiotic arm, to evaluate the potential confounding effect of ethnicity on results.

		ITT (n=42)				
**Measure**		**Interaction estimate (95%CI)**	**t value**	**p value**	**Cohen's d (95% CI)**	**Corrected Cohen's d**
**HAMD**	Week 4	2.58 (0.18-4.98)	2.17	0.04	0.94 (0.06-1.82)	0.90
	Week 8	1.89 (-0.86, 4.45)	1.49	0.15	0.69 (-0.25, 1.62)	0.66
**IDS-SR**	Week 4	4.55 (-0.66, 9.77)	2.02	0.09	0.61 (-0.09, 1.32)	0.59
	Week 8	6.03 (0.63, 11.44)	2.25	0.03	0.81 (0.08, 1.54)	0.78
**HAMA***	Week 4	0.58 (0.12, 1.04)	2.57	0.01	0.99 (0.21, 1.76)	0.95
	Week 8	0.25 (0.05, 104)	2.21	0.03	0.42 (0.08, 1.77)	0.40
**GAD**	Week 4	2.09 (-0.76, 4.95)	1.48	0.15	0.52 (-0.19, 1.22)	0.49
	Week 8	1.03 (-1.69, 3.74)	0.77	0.45	0.25 (-0.42, 0.92)	0.24

© 2023 Nikolova VL et al. *JAMA Psychiatry*.

eTable 4. Sensitivity Analysis by Non-SSRI Use

Estimates of treatment effect on depressive and anxiety symptoms after the removal of all participants taking antidepressants other than SSRIs (n=4), to evaluate the impact of treatment as potential confounder.

Measure		ITT (n=45) Interaction estimate (95%CI)	t value	p value	Cohen's d (95% CI)	Corrected Cohen's d
**HAMD**	Week 4	2.59 (0.49, 4.70)	2.48	0.02	0.82 (0.15, 1.48)	0.78
	Week 8	2.04 (-0.33, 4.42)	1.73	0.09	0.64 (-0.10, 1.39)	0.62
**IDS-SR**	Week 4	4.67 (-0.14, 9.47)	2.30	0.06	0.53 (-0.02, 1.07)	0.51
	Week 8	5.70 (0.50, 10.90)	2.21	0.03	0.64 (0.06, 1.23)	0.62
**HAMA***	Week 4	0.45 (0.06, 0.84)	2.34	0.02	0.74 (0.10-1.38)	0.71
	Week 8	0.59 (0.18, 1.01)	2.90	0.01	0.97 (0.30, 1.67)	0.93
**GAD**	Week 4	2.51 (-0.18, 5.19)	1.88	0.07	0.57 (-0.04, 1.19)	0.55
	Week 8	1.60 (-1.12, 4.32)	1.19	0.24	0.37 (-0.36, 0.99)	0.35

© 2023 Nikolova VL et al. *JAMA Psychiatry*.

eTable 5. Estimates of Treatment Effect on Depressive and Anxiety Symptoms in PP Dataset

Measure		Per Protocol (n=44) Interaction estimate (95%CI)	t value	p value	Cohen's d (95% CI)	Corrected Cohen's d
**HAMD**	Week 4	2.37 (0.17, 4.57)	2.17	0.04	0.76 (0.05, 1.47)	0.73
	Week 8	1.78 (-0.56, 4.12)	1.53	0.13	0.57 (-0.18, 1.33)	0.55
**IDS-SR**	Week 4	4.82 (-0.14, 9.77)	1.96	0.06	0.57 (-0.02, 1.16)	0.55
	Week 8	6.19 (1.07, 11.31)	2.44	0.02	0.73 (0.13, 1.34)	0.70
**HAMA***	Week 4	0.43 (-0.01, 0.86)	1.97	0.06	0.70 (-0.01, 1.41)	0.67
	Week 8	0.53 (0.09, 0.97)	2.44	0.02	0.87 (0.15, 1.59)	0.84
**GAD**	Week 4	2.47 (-0.25, 5.18)	1.83	0.07	0.70 (-0.01, 1.41)	0.56
	Week 8	1.79 (-0.82, 4.39)	1.38	0.17	0.42 (-0.19, 1.04)	0.40

* Interaction estimates based on sqrt-transformed values due to non-normally distributed data and residuals; interaction estimates show the time*group mean difference with positive values indicating a larger improvement in the probiotic group.

© 2023 Nikolova VL et al. *JAMA Psychiatry*.

eTable 6. IDS Items Included in the Rush (1966) Anxiety/Arousal Subscale

IDS item	Anxiety/arousal subscale
1. Initial insomnia	
2. Middle insomnia	
3. Early morning awakening	
4. Sleeping too much	
5. Feeling sad	
6. Feeling irritable	x
7. Feeling anxious or tense	x
8. Reactivity of mood	
9. Diurnal variation of mood	
10. Quality of mood	
11+12. Appetite disturbance	
13+14. Weight disturbance	
15. Concentration/decision-making	
16. Self criticism and blame	
17. Future pessimism	
18. Suicidal thoughts	
19. Interest in people/activities	
20. Energy/fatigability	
21. Pleasure or enjoyment (not sex)	
22. Interest in sex	
23. Psychomotor retardation	x
24. Psychomotor agitation	x
25. Aches and pains	x
26. Sympathetic arousal	x
27. Panic/phobic symptoms	x
28. Constipation/diarrhoea	x
29. Interpersonal sensitivity	
30. Leaden paralysis/physical energy	x

© 2023 Nikolova VL et al. *JAMA Psychiatry*.

eReferences.

1. American Psychiatric Association, American Psychiatric Association DSM-5 Task Force. *Diagnostic and Statistical Manual of Mental Disorders: DSM-5.* 5th ed.. American Psychiatric Association; 2013.

2. Nikolova VL, Cleare AJ, Young AH, Stone JM. Updated Review and Meta-Analysis of Probiotics for the Treatment of Clinical Depression: Adjunctive vs. Stand-Alone Treatment. *J Clin Med*. 2021;10(4). doi:10.3390/jcm10040647

3. Schaub AC, Schneider E, Vazquez-Castellanos JF, et al. Clinical, gut microbial and neural effects of a probiotic add-on therapy in depressed patients: a randomized controlled trial. *Transl Psychiatry*. 2022;12(1):1-10. doi:10.1038/s41398-022-01977-z

4. Yong SJ, Tong T, Chew J, Lim WL. Antidepressive Mechanisms of Probiotics and Their Therapeutic Potential. *Front Neurosci*. 2020;13:1361. doi:10.3389/fnins.2019.01361

5. Baião R, Capitão LP, Higgins C, Browning M, Harmer CJ, Burnet PWJ. Multispecies probiotic administration reduces emotional salience and improves mood in subjects with moderate depression: a randomised, double-blind, placebo-controlled study. *Psychological Medicine*. Published online February 7, 2022:1-11. doi:10.1017/S003329172100550X

6. Rahimlou M, Hosseini SA, Majdinasab N, Haghighizadeh MH, Husain D. Effects of long-term administration of Multi-Strain Probiotic on circulating levels of BDNF, NGF, IL-6 and mental health in patients with multiple sclerosis: a randomized, double-blind, placebo-controlled trial. *Nutr Neurosci*. 2022;25(2):411-422. doi:10.1080/1028415X.2020.1758887

7. Factsheet: The reasoning behind the Bio-Kult dosage. Published online November 2017. Accessed November 26, 2020. https://www.bio-kult.com/userfiles/file/m0034-02_2457_bio-kult_dosage_factsheet_update_lr.pdf

8. Tejero-Sariñena S, Barlow J, Costabile A, Gibson GR, Rowland I. In vitro evaluation of the antimicrobial activity of a range of probiotics against pathogens: evidence for the effects of organic acids. *Anaerobe*. 2012;18(5):530-538. doi:10.1016/j.anaerobe.2012.08.004

9. Svedlund J, Sjödin I, Dotevall G. GSRS—A clinical rating scale for gastrointestinal symptoms in patients with irritable bowel syndrome and peptic ulcer disease. *Digest Dis Sci*. 1988;33(2):129-134. doi:10.1007/BF01535722

10. Heck RH, Thomas S, Tabata L. *Multilevel Modeling of Categorical Outcomes Using IBM SPSS*. Taylor & Francis Group; 2012. Accessed September 7, 2022. http://ebookcentral.proquest.com/lib/kcl/detail.action?docID=1092838

© 2023 Nikolova VL et al. *JAMA Psychiatry*.
