# Supplementary material for: Acceptability, Tolerability, and Estimates of Putative Treatment Effects of Probiotics as Adjunctive Treatment in Patients With Depression: A Randomized Clinical Trial
Source: JAMA Psychiatry. 2023 Jun 14;80(8):842–7. doi: 10.1001/jamapsychiatry.2023.1817 (PMC10267847; doi:10.1001/jamapsychiatry.2023.1817)
Supplement: Supplement 3. — Data Sharing Statement [file jamapsychiatry-e231817-s003.pdf]

Data Sharing Statement

Nikolova. Acceptability, Tolerability, and Estimates of Putative Treatment Effects of Probiotics as Adjunctive Treatment in Patients With Depression. *JAMA Psychiatry*. Published June 14, 2023. doi:10.1001/jamapsychiatry.2023.1817

Data
Data available: Yes
Data types: Deidentified participant data, Data dictionary
How to access data: Data will be made available upon reasonable request, please email j.stone@bsms.ac.uk.
When available: With publication

Supporting Documents
Document types: None

Additional Information
Who can access the data: researchers whose proposed use of the data has been approved
Types of analyses: Specified approved purposes
Mechanisms of data availability: with a signed data access agreement
